# Diversity in new flagellum tip attachment in bloodstream form African trypanosomes

**DOI:** 10.1111/mmi.14979

**Published:** 2022-09-14

**Authors:** Laura Smithson, Pearl Ihuoma Akazue, Lucy Findlater, Theresa Manful Gwira, Sue Vaughan, Jack D. Sunter

**Affiliations:** ^1^ Department of Biological and Medical Sciences Oxford Brookes University Oxford UK; ^2^ West African Centre for Cell Biology of Infectious Pathogens, College of Basic and Applied Sciences University of Ghana Accra Ghana; ^3^ Department of Biochemistry, Cell and Molecular Biology, College of Basic and Applied Sciences University of Ghana Accra Ghana; ^4^ Department of Biochemistry, Faculty of Life Sciences University of Benin Benin City Nigeria; ^5^ Sir William Dunn School of Pathology University of Oxford Oxford UK

**Keywords:** Flagellum, morphogenesis, serial block face scanning electron microscopy (SBF‐SEM), trypanosomes

## Abstract

The closely related parasites *Trypanosoma brucei*, *T. congolense*, and *T. vivax* cause neglected tropical diseases collectively known as African Trypanosomiasis. A characteristic feature of bloodstream form *T. brucei* is the flagellum that is laterally attached to the side of the cell body. During the cell cycle, the new flagellum is formed alongside the old flagellum, with the new flagellum tip embedded within a mobile transmembrane junction called the groove. The molecular composition of the groove is currently unknown, which limits the analysis of this junction and assessment of its conservation in related trypanosomatids. Here, we identified 13 proteins that localize to the flagellar groove through a small‐scale tagging screen. Functional analysis of a subset of these proteins by RNAi and gene deletion revealed three proteins, FCP4/TbKin15, FCP7, and FAZ45, that are involved in new flagellum tip attachment to the groove. Despite possessing orthologues of all 13 groove proteins, *T. congolense* and *T. vivax* did not assemble a canonical groove around the new flagellum tip according to 3D electron microscopy. This diversity in new flagellum tip attachment points to the rapid evolution of membrane‐cytoskeleton structures that can occur without large changes in gene complement and likely reflects the niche specialization of each species.

## Graphical abstract

Using 3D‐electron microscopy, we have found that the flagellar groove represents a specific adaptation of *Trypanosoma brucei*, with this structure absent in *Trypanosoma congolense* and *Trypanosoma vivax*. Furthermore, through an endogenous tagging screen, we have successfully identified 13 proteins that localise to the *Trypanosoma brucei* flagellar groove. Several of these proteins are involved in new flagellum tip attachment to the groove and cytokinesis.

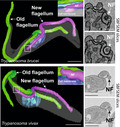

## 1 INTRODUCTION

African trypanosomiasis is a vector‐borne neglected tropical disease caused by parasites of the *Trypanosoma* genus that are cyclically transmitted through the bite of an infected tsetse fly. Two forms of the disease are endemic to sub‐Saharan Africa: Human African Trypanosomiasis, also known as sleeping sickness, and Animal African Trypanosomiasis, commonly known as *nagana* (Aksoy et al., 2014). *Nagana*, caused by *Trypanosoma brucei*, *Trypanosoma vivax*, and *Trypanosoma congolense*, is considered to be the main disease that limits the trade of livestock in sub‐Saharan Africa and kills approximately 3 million cattle annually, with an estimated loss of US $4.5 billion (Giordani et al., 2016; Hamill et al., 2013; Schofield & Kabayo, 2008).

The African trypanosomes, *T. brucei*, *T. vivax*, and *T. congolense*, have complex life cycles alternating between the mammalian host and the insect vector. However, despite the similarities in their life cycles, these parasites have different ecological niches within their vectors and hosts (Reviewed in Pereira et al., 2019). Furthermore, distinct differences in the overall morphology and motility of these parasites have been documented previously (Bargul et al., 2016). The vast majority of current research efforts have focused on *T. brucei*, which is considered a paradigm for the other African trypanosomes. The shape of a *T. brucei* trypomastigote cell is defined by a highly structured array of subpellicular microtubules and the position of certain single copy organelles, such as the nucleus, kinetoplast (concatenated mitochondrial DNA), basal body, and flagellum (Gull, 1999; Sherwin & Gull, 1989; Vickerman, 1969). In *T. brucei*, the flagellum is essential for cell morphogenesis, motility, and pathogenicity (Bastin et al., 1998; Bastin et al., 2000; Griffiths et al., 2007). The basal body nucleates the flagellum, which then extends out of the flagellar pocket towards the posterior end of the cell and is laterally attached to the side of the cell body for the majority of its length through the flagellum attachment zone (FAZ). The FAZ is a large, complex, cytoskeletal structure that connects the cytoskeleton through the cell body and flagellum membranes to the flagellar cytoskeleton (Sherwin & Gull, 1989). Many FAZ proteins have been identified by our group and other groups; however, not all are distributed evenly across the structure, including a subset restricted to the distal end of the FAZ (An & Li, 2018; Dean et al., 2017; Sunter, Benz, et al., 2015; Sunter & Gull, 2016; Sunter, Varga, et al., 2015; Vaughan et al., 2008).

The fidelity of cell morphogenesis between successive generations of parasites is critical. Cell division in *T. brucei* involves both semi‐conservative (e.g., subpellicular microtubule array) and conservative (e.g., flagellum, FAZ, basal body) inheritance patterns (Gull, 1999; Wheeler et al., 2019). Several differences in organelle structure and biochemical adaptations exist between the two most studied proliferative forms of *T. brucei*: the procyclic form (PCF) and the bloodstream form (BSF) (Wheeler et al., 2013). During cell division, a PCF cell nucleates a new flagellum from the pro‐basal body, which exits the existing flagellar pocket (FP) to grow alongside the old flagellum. The new flagellum tip is physically attached to the old flagellum by the flagella connector, a mobile transmembrane junction that connects the two flagella (Briggs et al., 2004; Davidge et al., 2006; Moreira‐Leite et al., 2001). The flagella connector migrates along the old flagellum as the new flagellum elongates, and once the new flagellum is ~0.6 of the length of the old flagellum, the flagella connector reaches a “stop‐point,” with a division fold forming between the two flagella (Davidge et al., 2006). The flagella connector maintains the connection between the new flagellum tip and the old flagellum as cytokinesis initiates (Briggs et al., 2004; Davidge et al., 2006; Moreira‐Leite et al., 2001). Cytokinesis requires the progressive recruitment of multiple proteins such as CIF1‐4 and FPRC to the distal tip of the new FAZ. The loss of each of these proteins individually results in a severe cytokinesis defect (Hilton et al., 2018; Hu et al., 2019; Kurasawa et al., 2018; McAllaster et al., 2015; Sinclair‐Davis et al., 2017; Zhou et al., 2016; Zhou et al., 2018). Finally, the flagella connector releases the new flagellum distal tip, and the abscission of the membrane joining the two posterior ends separates the two daughter cells (Moreira‐Leite et al., 2001).

The flagella connector is a trilaminar cytoskeletal structure composed of three distinct electron‐dense layers found across the new flagellum membrane, the old flagellum membrane, and the intermediate space between the membranes (Höög et al., 2016). Seven proteins have been identified that localize to specific zones in the flagella connector, and the depletion of four of these proteins (FCP1/2/4 and FC1) resulted in the premature severing of the connection between the new flagellum tip and the side of the old flagellum (McAllaster et al., 2015; Varga et al., 2017).

BSF cells follow a similar pattern of organelle duplication and segregation as the PCF; however, during new flagellum growth, the new flagellum tip is not tethered to the side of the old flagellum by the flagella connector. Instead, the new flagellum tip is embedded in a discrete indentation of the cell body plasma membrane, and this complex of indentation and flagellum tip is termed the groove (Hughes et al., 2013). The groove is a mobile transmembrane junction that migrates beside the old flagellum as the new flagellum elongates, initiating significant remodeling of the subpellicular microtubule array during its migration, with microtubules positioned both to the anterior and posterior of the structure (Hughes et al., 2013). Once the groove approaches the anterior cell tip, the groove resolves and releases the distal tip of the new flagellum prior to cytokinesis.

The groove is a uniquely differentiated area of the plasma membrane, forming a transmembrane junctional complex composed of two membranes of different compositions. Punctate and filamentous electron‐dense material underlie the cytoplasmic face of the cell body plasma membrane of the groove structure, potentially involved in mediating flagellum tip attachment in the groove (Hughes et al., 2013). Furthermore, filament connections across both the flagellum and cell body plasma membranes in the groove were seen by thin‐section TEM. These structures resembled the FAZ connections that attach the flagellum to the cell body, but despite this resemblance, the groove was not labeled with an antibody that recognizes the canonical FAZ filament (Hughes et al., 2013). Interestingly, DOT1, which labels the punctate junctional complexes of the FAZ, localized to the flagellar groove as an elaboration at the distal end of the new flagellum (Hughes et al., 2013; Woods et al., 1989). This supports the idea that the groove filament network, despite sharing some biochemical similarities with the FAZ, is not wholly just an extension of the FAZ but an elaboration of this structure.

The molecular components contributing to the formation, migration, and resolution of the groove are mostly unknown. We used the PCF genome‐wide localization database, TrypTag, to identify putative BSF groove proteins by selecting proteins that localized to the flagella connector and distal FAZ/anterior cell tip in the PCF (Dean et al., 2017). Here, we identified 13 groove proteins in the BSF through PCR‐based endogenous tagging. Overall, there was limited overlap between the flagella connector and groove, pointing to life cycle‐specific elaboration of these structures. The loss of groove proteins resulted in cytokinesis defects and/or mistiming of the release of the new flagellum tip. Interestingly, despite the conservation of these groove proteins in the different African trypanosomes, we found species‐specific innovation of the cytoskeletal structures at the tip of the growing new flagellum that likely reflects the different ecological niches these parasites occupy in their host and vector.

## 2 RESULTS

### 2.1 Flagellar groove resolves post‐mitosis

**FIGURE 1:**
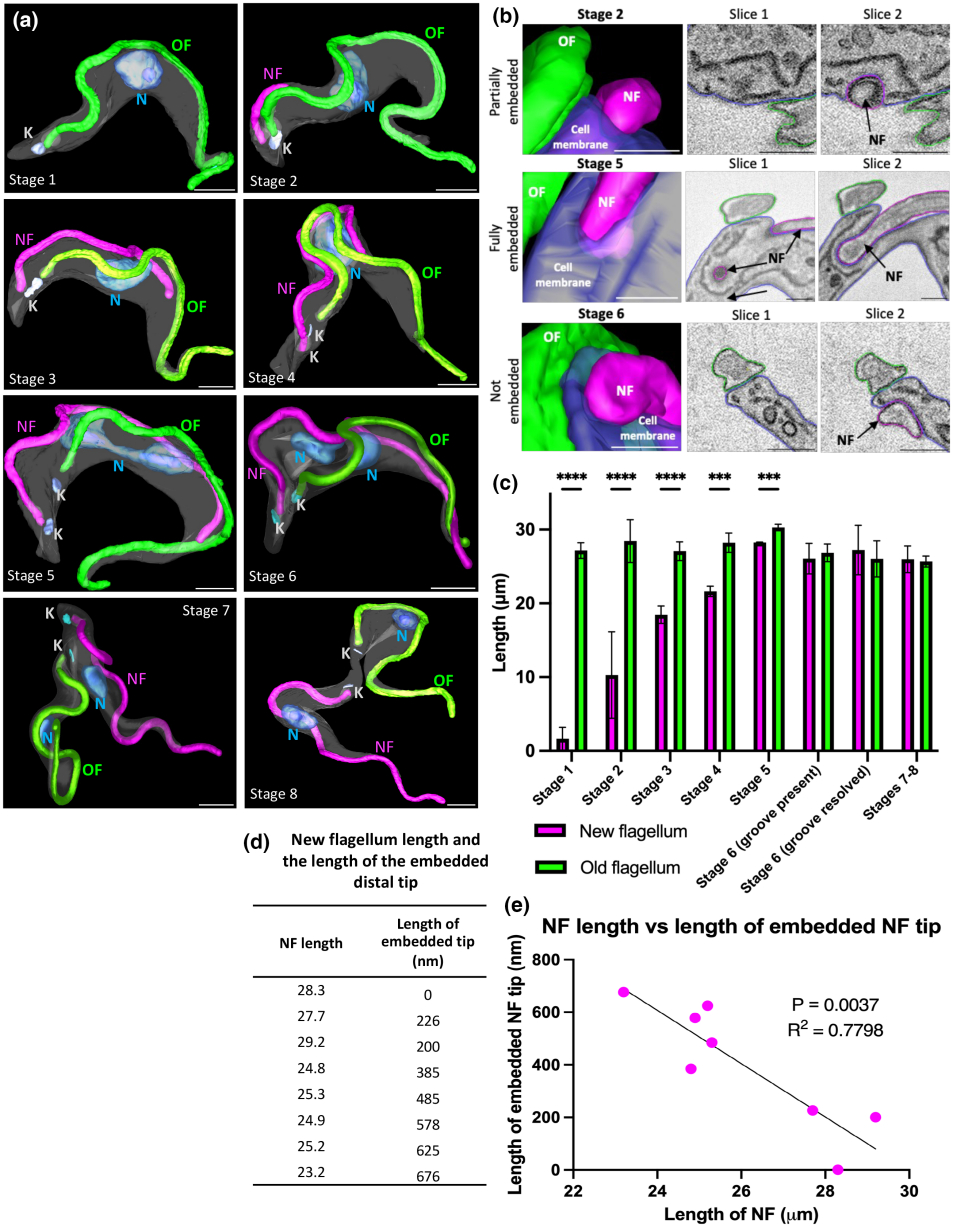
Timing of groove resolution post‐mitosis correlates with a new flagellum that is longer than the old flagellum. (a) 3D surface rendering of representative cells during stages 1–8. Stage 1: G1 cell with one kinetoplast, one nucleus, and a single flagellum; Stage 2: Cell with a short new flagellum alongside the old flagellum, with the nucleus increasing in volume and the new flagellum distal tip partially embedded in the cell membrane; Stage 3: Cell with two kinetoplasts, one large nucleus, and nucleolus and a long new flagellum with the distal tip fully embedded in the groove; Stage 4: Cell with two kinetoplasts and one large nucleus (containing a dividing nucleolus), with a long new flagellum; Stage 5: Cell containing two kinetoplasts and a dividing nucleus, with a long new flagellum; Stage 6: Cell containing two kinetoplasts and two nuclei, each containing a small nucleolus. At this stage, the groove has resolved and released the new flagellum distal tip from the cell body; Stage 7–8: Cells undergoing cell division. Key: K = kinetoplast; N = nucleus; NF = new flagellum; OF = old flagellum. Scale = 2 μm. (b) Enlarged regions of the new flagellum tip in representative cells from stages 2, 5, and 6 showing the change in depth of new flagellum tip embedded in the groove. Representative images do not necessarily correspond to images from (a). Surface rendering of the new flagellum tip region alongside SBF‐SEM raw data slices of the modeled area moving from distal to proximal. Scale = 500 nm. (c) Mean length of the new and old flagella during stages 1–8. The number of cells modeled and measured per cell cycle stage are as follows: Stage 1 = 3; Stage 2 = 4; Stage 3 = 4; Stage 4 = 3; Stage 5 = 4; Stage 6 = 22; Stage 7 = 5. Three independent SBF‐SEM data sets were used to model cells in the defined cell cycle stages. Error bars show the standard deviation of the mean length of the new and old flagella per cell cycle stage (±*SD*). ****p < .0001; ***p < .005 by Unpaired Student's *t* test. (d) Table of the length of the new flagellum in 2K2N cells from stage 6 showing the change in depth of the embedded new flagellum tip. (e) Graphical representation of data from (d) showing the correlation between the length of the new flagellum and the depth of the embedded new flagellum tip.

In our previous work, we discovered that the flagellar groove migrated along the cell body during progression of the cell cycle (Hughes et al., 2013). Here, we sought to investigate flagellar groove resolution in greater detail. To investigate the timing of resolution, we used SBF‐SEM to reconstruct 45 individual cells across the cell cycle from four independent SBF‐SEM runs. The cells were grouped into seven distinct cell cycle stages, modified from Hughes et al. (2017), with cells undergoing cleavage furrow ingression and cytokinesis grouped into a single cell cycle stage (stage 7–8) (Figure 1a). The cell cycle started with a G1 cell containing one kinetoplast, one nucleus, and a single flagellum (Figure 1a stage 1). Next, a new flagellum emerged from the flagellar pocket (Figure 1a stage 2), with the new flagellum distal tip partially embedded in the cell body plasma membrane (Figure 1b). As the kinetoplast is duplicated (Figure 1a stage 3), the new flagellum tip was fully embedded in a deep groove in the cell body plasma membrane through mitosis (Figure 1a,b stages 4 and 5). In post‐mitotic cells (Figure 1a stage 6), only 36% (*N* = 22) possessed a groove, with the new flagellum tip free from the cell body in the remaining 64% (Figure 1a,b stage 6), suggesting that the groove resolves post‐mitosis.

During stages 1–5, the new flagellum was always shorter than the old flagellum and was positioned in the flagellar groove (Figure 1c). We found that the average length of the new flagellum (27.2 μm) in stage 6 with a resolved groove was actually longer than the average length of the old flagellum (26 μm); however, this difference was not statistically significant for the sample size used in this study (Figure 1c). In stages 7–8, when cytokinesis is underway, the new flagellum shortens until it is essentially the same length as the old flagellum.

Groove resolution is likely associated with a decrease in the length to which the new flagellum tip is embedded. To investigate this, we analyzed the eight stage 6 cells in which the new flagellum tip was still associated with the cell body (Figure 1d,e) and found that, interestingly, the decrease in the length of the embedded tip was associated with the length of the new flagellum exceeding that of the old flagellum (Figure 1d,e). In five cells, the new flagellum tip was embedded for 385–676 nm (Figure 1d). For the three remaining cells, there was a clear decrease in the length of embedded tip, with one of these cells possessing a new flagellum with the tip associated with a cell body indentation but was clearly not embedded (as seen in Figure 1b stage 6). Moreover, in these eight stage 6 cells, the nearest the groove approached the anterior cell tip was 5.6 μm and this was in the cell with a notably shallow groove (Figure 1b stage 6). This suggests that the groove resolves a distance from the anterior cell tip by a gradual shallowing.

### 2.2 Identification of 13 flagellar groove proteins

We used the TrypTag database to identify proteins with the potential to localize to the flagellar groove. This method has an evident caveat of using the PCF localization database to identify proteins that localize to the groove in the BSF. However, as the groove is associated with the new FAZ and likely associated with the new anterior cell tip, proteins localizing to these areas in the PCF were selected. Our candidate selection was further refined to 45 proteins by only including those with a clear, visible signal at the distal FAZ or anterior cell tip. In addition, all identified flagellar connector proteins were included (Varga et al., 2017).

**FIGURE 2:**
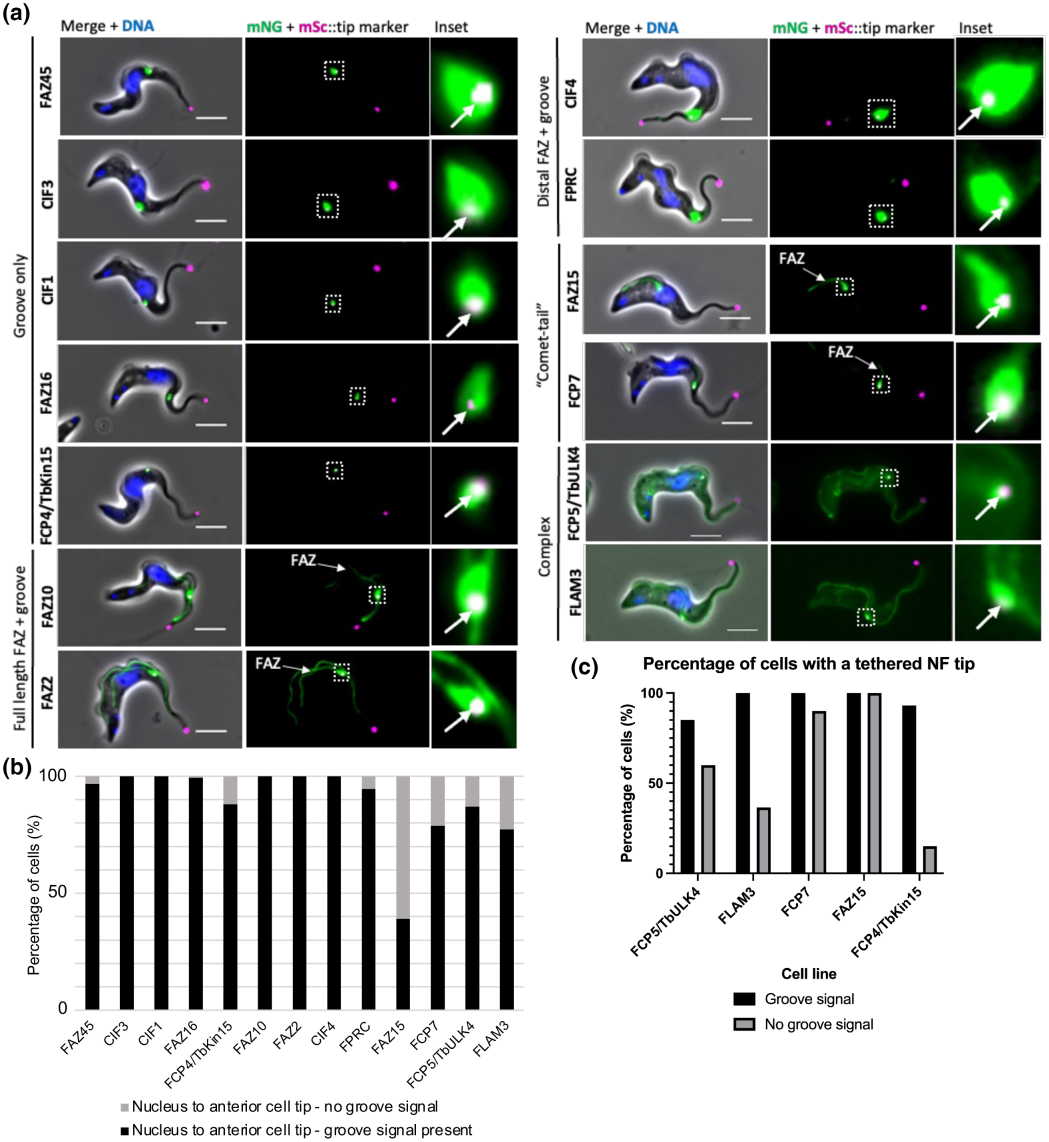
13 proteins localize to the flagellar groove with five distinct localization patterns. (a) Representative images of the endogenous tagging of the candidate groove proteins with mNeonGreen revealed 13 proteins that localize to the flagellar groove as a distinct elaboration around the new flagellum distal tip (mSc::Tb427.02.5860) seen in the inset box. Scale = 5 μm. (b) Presence and absence of the groove signal between the nucleus and anterior cell tip. (*N* = 500 2F cells where the new flagellum tip marker is clearly visible). (c) Percentage of cells with or without a groove signal that maintain a tethered new flagellum distal tip. (*N* = 30 cells in which the new flagellum tip is located between the anterior edge of the nucleus and the anterior cell tip).

A marker cell line expressing the flagellum tip protein (Tb427.02.5860) fused to the mScarlet (mSc) fluorescent protein was used to help identify flagellar groove proteins (Figure S1). In 1K1N cells, mSc::Tb427.02.5860 localized to the distal tip of the flagellum and, as the new flagellum began to grow, also localized to the new flagellum tip (Figure S1b). We saw that the signal intensity of the flagellum tip marker was weaker on the tip of the new versus the old flagellum (Figure S1b–f) and of the 1K1N cells with a visible signal on the new flagellum, all possessed a bilobed kinetoplast, suggesting that the marker was only visible on the new flagellum tip after the cell entered kinetoplast S‐phase. Each groove protein candidate was tagged with mNeonGreen (mNG) in the marker cell line and examined using widefield fluorescence microscopy. A groove protein was defined as having a clear, visible elaboration of the mNG signal co‐localizing around the tip of the new flagellum. Of the 45 proteins screened, only 13 localized to the groove and these proteins were subsequently classified into five categories (Figures 2a and S2): (1) Tb427tmp.01.3030 (FAZ45), Tb427.10.13100 (CIF3), Tb427tmp.01.7450 (CIF1), Tb427.05.3460 (FAZ16), and Tb427.10.890 (FCP4/TbKin15) all localized exclusively to the groove during new flagellum growth; (2) Tb427.07.3330 (FAZ10) and Tb427.01.4310 (FAZ2) localized to the flagellar groove and the full length of both the old and new FAZ; (3) Tb427.10.6360 (FPRC) and Tb427.10.8240 (CIF4) localized to the groove, with an additional foci observed along a portion of the distal end of the old FAZ; (4) Tb427.08.7070 (FAZ15) and Tb427.01.2710 (FCP7) localized to a portion of the new FAZ and to the groove, resembling a “comet‐tail” during new flagellum growth; (5) Tb427tmp.01.0400 (FCP5/TbULK4) and Tb427.08.4780 (FLAM3) localized to the groove and several other areas of the cell forming a complex localization pattern. In cells growing a new flagellum, mNG::FCP5/TbULK4 localized to the groove, flagellum, basal body pair, cytoplasm, and nucleoplasm (Figure 2a). In contrast, in cells growing a new flagellum, mNG::FLAM3 localized to the groove, FAZ, and cytoplasm (Figure 2a). The localization screen identified 32 proteins that did not localize to the flagellar groove as the mNG signal did not fit our criteria (Figure S3).

### 2.3 Groove proteins have cell cycle‐dependent localization patterns

Our earlier SBF‐SEM analysis indicated that groove resolution occurred post‐mitosis, with resolution occurring when the new flagellum tip is located between the anterior edge of the nucleus and the anterior cell tip. The presence and absence of the groove proteins were scored in cells with a new flagellum tip observed between the anterior edge of the nucleus and the anterior cell tip to further understand the dynamics of groove protein localization and groove resolution (Figures 2b and S4a). We observed no apparent differences in timing of appearance of the groove proteins but there were differences in the timing of the disappearance of these proteins (Figure 2b). The proportion of cells seen without a groove signal indicates the point in the cell cycle at which that protein likely disappears—the larger the proportion of cells without a groove signal the earlier in the cell cycle this protein is lost and vice versa. Overall, we saw four different patterns. Firstly, for mNG::FAZ10, mNG::CIF4, mNG::CIF3, CIF1::mNG, and mNG::FAZ2, the position analysis confirmed that these proteins were present between the nucleus and anterior cell tip in all cells (Figure 2b). In addition, mNG::CIF4, mNG::CIF3, and CIF1::mNG localized to the cleavage furrow in cells undergoing cytokinesis (Figure S4b). Secondly, the mNG::FPRC, mNG::FAZ16, and mNG::FAZ45 signals were present between the nucleus and anterior cell tip in the vast majority of cells (≥94%) examined; however, these proteins disappeared prior to cytokinesis initiation and were not present in the cleavage furrow (Figure 2b). Thirdly, the mNG::FCP4/TbKin15, mNG::FCP5/TbULK4, FCP7::mNG, and mNG::FLAM3 signals were not present in 12%–23% of cells in which the new flagellum tip was present between the nucleus and anterior cell tip (Figure 2b). Fourthly, the mNG::FAZ15 signal was not present in ~60% of this cell type, suggesting that this protein may even disappear from the groove before it has resolved (Figure 2b).

The disappearance of certain proteins during the cell cycle may be linked to groove resolution. To determine whether protein disappearance coincided with groove resolution, we analyzed the proportion of cells in the FCP5/TbULK4, FLAM3, FCP7, FAZ15, and FCP4/TbKin15 tagged cell lines with and without a groove signal that still had a tethered new flagellum tip (Figure 2c). For mNG::FCP5/TbULK4 and mNG::FLAM3, there was no clear pattern seen. 85% and 100% of cells (*N* = 30) with a groove signal still had a tethered new flagellum tip, respectively (Figure 2c). Once the groove signal disappeared, 60% and 36.6% of cells (*N* = 30) maintained an attached new flagellum tip (Figure 2c). While, for the FCP7::mNG and mNG::FAZ15 cell lines, 100% of cells (*N* = 30) with a groove signal still possessed a tethered new flagellum tip across both cell lines and even when the groove signal of these proteins disappeared, the new flagellum tip was tethered in 90% and 100% of cells (*N* = 30), respectively (Figure 2c). This shows that FCP7 and FAZ15 protein disappearance occurs before groove resolution. Conversely, for mNG::FCP4/TbKin15, 93% of cells (*N* = 30) with a groove signal still had a tethered new flagellum tip; however, once the groove signal disappeared, only 15% of cells (*N* = 30) had a tethered new flagellum tip, suggesting that FCP4/TbKin15 disappearance occurred around groove resolution (Figure 2c).

### 2.4 Groove is composed of proteins required for new flagellum tip attachment and cytokinesis

We chose to focus our functional analysis on groove proteins that had a restricted localization to the groove and distal FAZ and had not previously been examined in BSF *T. brucei*. For our initial approach, we used CRISPR/Cas9‐mediated gene deletion to generate deletion cell lines for CIF4, FAZ45, FCP7, FAZ15, and FAZ16 in the background of the flagellum tip marker cell line. We were unable to generate deletion lines for FCP4/TbKin15 and FPRC despite multiple attempts. Loss of the expected open reading frame (ORF) was analyzed by PCR to confirm the deletion of CIF4, FAZ45, FCP7, FAZ15, and FAZ16, and one successful clone of each gene deletion was analyzed (Figure S5).

**FIGURE 3:**
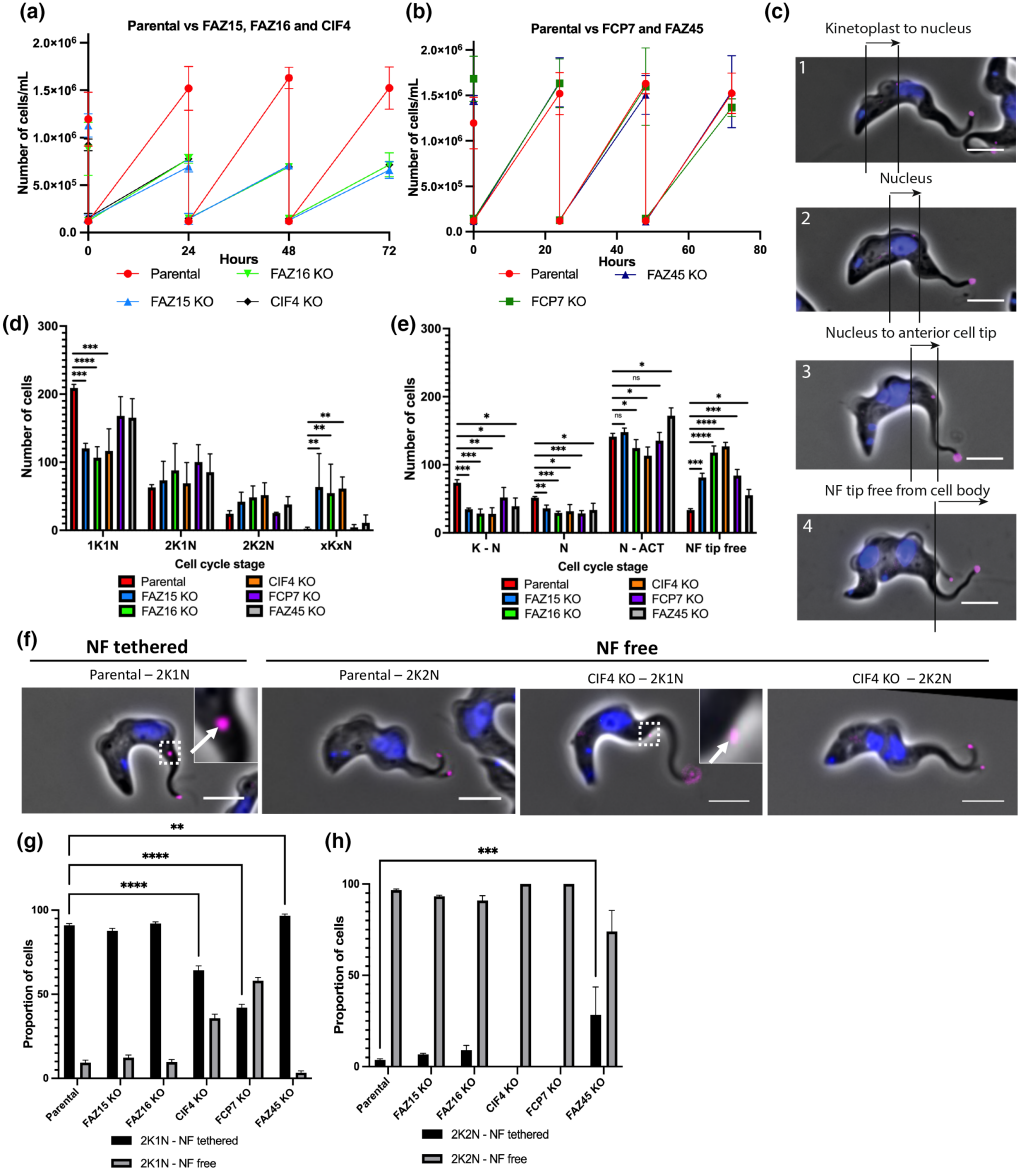
Deletion of FCP7 and CIF4 caused premature detachment of the new flagellum tip from the groove. (a and b) Growth curves of the deletion cell lines generated through CRISPR/Cas‐9. Cell lines with a clear growth defect are shown in (a), whereas cell lines with no growth defect are shown in (b). (c) Example images of the defined zones used to analyze the position of the new flagellum distal tip. (d) Analysis of the number of kinetoplasts and nuclei in the deletion cell lines compared to the parental cell line. Error bars show standard deviation of the mean of 3 independent technical replicates (*n* = 300 cells per replicate). ****p < .0001; ***p < .001; **p < .01; *p < .05 by Unpaired Student's *t* test. (e) Position analysis of the deletion cell lines examining the position of the new flagellum distal tip (tagged with mSc::Tb427.02.5860) along the cell body. Error bars show standard deviation of the mean of 3 independent technical replicates (*n* = 300 cells with a visible new flagellum tip signal). ****p < .0001; ***p < .001; **p < .01; *p < .05 by Unpaired Student's *t* test. (f) Representative images of the data in (g) showing the new flagellum still associated with the cell body in parental 2K1N cells. The new flagellum tip is free from the cell body in parental 2K2N cells but also in the CIF4 KO 2K1N and 2K2N cells, suggesting that the deletion of CIF4 causes premature new flagellum tip detachment from the groove. (g, h) Analysis of the proportion of 2K1N and 2K2N cells with a tethered and free new flagellum distal tip. Error bars show standard deviation of the mean of 3 independent technical replicates (*n* = 100 cells per replicate). ****p < .0001 by two‐way ANOVA with Šidák's multiple comparison test.

The deletion of FAZ15, CIF4, or FAZ16 caused a severe growth defect, while the FCP7 and FAZ45 deletion cell lines grew at a comparable rate to the parental cell line (Figure 3a,b). The effect of the loss of each of these proteins on the cell cycle was then examined by counting the number of kinetoplasts and nuclei present in each cell (Figure 3d). This indicates the cell cycle stage and allows us to assess cell cycle defects such as an accumulation of multinucleic cells and cells with an abnormal ratio of kinetoplasts to nuclei (“xKxN”). The deletion of FAZ15, CIF4, and FAZ16 each had a significant effect on the cell cycle, with a defect in cytokinesis characterized by a decrease in the proportion of 1K1N cells and increase in xKxN cells (Figure 3d). In comparison, deletion of FCP7 and FAZ45 each had a lesser effect on the cell cycle with a drop in 1K1N cells and a slight increase in the number of 2K2N and xKxN cells (Figure 3d).

To investigate whether the deletion of the genes encoding these groove proteins affected groove movement and/or resolution to release the new flagellum tip, we analyzed the position of the new flagellum tip along the cell body using the flagellum tip marker as a reference in cells with two flagella (Figure 3e). We categorized the cells into four distinct groups based on the position of the tip marker: (1) signal was visible between the kinetoplast and the posterior edge of the nucleus, (2) signal was visible adjacent to the nucleus, (3) signal was visible between the anterior edge of the nucleus and the anterior cell tip, and (4) tip signal was visible free from the cell body (Figure 3c). The deletion of FAZ15, CIF4, FAZ16, FCP7, and FAZ16 each caused a significant increase in the number of cells with a free new flagellum tip, whereas the deletion of FAZ45 had a less significant effect (Figure 3e). Our cell cycle analysis showed an increase in the number of cells in the later stages of the cell cycle for four of the deletion cell lines, which resulted in an increase in the overall number of cells seen with a free new flagellum tip. Therefore, we sought to examine the 2K1N and 2K2N cell cycle stages separately to determine whether there was premature release of the new flagellum tip from the groove. According to our SBF‐SEM analysis, the new flagellum tip should be fully embedded in the groove during mitosis; therefore, by quantifying the proportion of 2K1N cells with a free new flagellum tip, we can determine if the deletion caused premature new flagellum tip release (Figure 3f–h). The deletion of CIF4 and FCP7 each caused a significant increase in the number of 2K1N cells with a free new flagellum tip compared to the control cell line (*p* < .0001; two‐way ANOVA with Šidák's multiple comparison test) (Figure 3f–h = image 2K1N free tip). This suggests that deletion of CIF4 and FCP7 individually causes premature release of the new flagellum tip. However, deletion of FAZ15 or FAZ16 did not cause an increase in the number of 2K1N cells with a free new flagellum tip. Interestingly, the deletion of FAZ45 caused a decrease in the number of 2K2N cells with a free new flagellum tip in comparison to the parental cell line (*p* < .0001; two‐way ANOVA with Šidák's multiple comparison test) (Figure 3f–h). This suggests that deletion of FAZ45 caused a delay in the release of the new flagellum tip from the groove.

To confirm the effects of the CIF4, FCP7, or FAZ45 deletion on groove resolution, we examined these cells by SEM. In both the CIF4 and FCP7 deletion mutants, ~95% (*n* = 75) and ~ 70% (*n* = 160) of cells with two flagella had a free new flagellum tip, respectively, in comparison to ~23% (*n* = 321) of two flagella cells in the parental cell line (Figure S6). Importantly, in those cells that did not have a free flagellum tip, the tip was embedded in the cell body, suggesting the groove was still able to form despite the loss of CIF4 or FCP7. In the FAZ45 deletion cell line, the opposite effect was seen with an increased proportion of two flagella cells (86%; *n* = 122) possessing an embedded new flagellum tip in comparison to the parental cell line (77%; *n* = 321). This suggests that FAZ45 is required for the efficient release of the new flagellum tip from the groove.

### 2.5 Depletion of FCP4/TbKin15 resulted in premature new flagellum tip release from the groove

**FIGURE 4:**
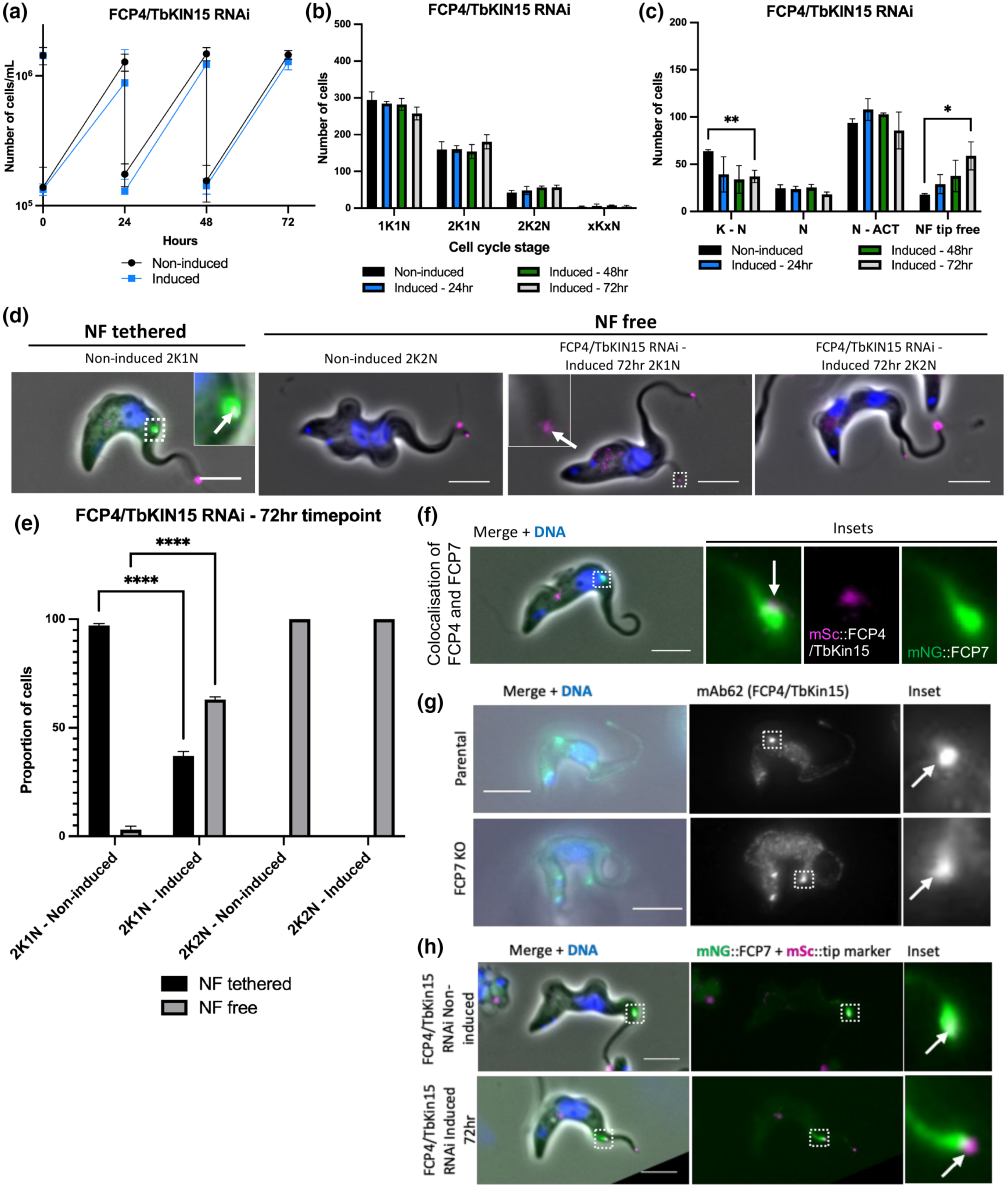
Depletion of FCP4/TbKin15 caused premature new flagellum tip detachment from the groove. (a) Growth curve of the FCP4/TbKin15 RNAi cell line. (b) Analysis of the number of kinetoplasts and nuclei in the FCP4/TbKin15 depletion cell line after induction with doxycycline. (*N* = 500 cells per timepoint). Unpaired Student's *t* test was used to compare the induced populations to the non‐induced control; however, the results were non‐significant. (c) Position analysis of the FCP4/TbKin15 RNAi cell line examining the position of the new flagellum distal tip (tagged with mSc::Tb427.02.5860) along the cell body. (*N* = 200 cells per timepoint with a visible new flagellum tip signal). *p < .05 by Unpaired Student's *t* test. (d) Representative images of the data in (e) showing the new flagellum still associated with the cell body in the non‐induced 2K1N cells. The new flagellum tip is free from the cell body in the non‐induced 2K2N cells but also in the induced 2K1N and 2K2N cells, suggesting that the depletion of FCP4/TbKin15 causes premature new flagellum tip detachment from the groove. (e) Analysis of the proportion of 2K1N and 2K2N cells with a tethered and free new flagellum distal tip. Error bars show standard deviation of the mean of 3 independent technical replicates (*n* = 100 cells per replicate). ****p < .0001 by two‐way ANOVA. (f) Colocalization of mNG::FCP7 and mSc::FCP4/TbKin15. (g) Presence of mAb62 signal in the parental and FCP7 deletion cell line. (h) Localization of mNG::FCP7 in the FCP4/TbKin15 RNAi cell line. mSc::tip marker present on the new and old flagella tips. Scale = 5 μm.

Given the importance of FCP4/TbKin15 in maintaining the connection between the new and old flagellum in PCFs (Varga et al., 2017), we investigated its function in BSFs using an inducible RNAi approach. A plasmid encoded with a hairpin RNAi construct targeting FCP4/TbKin15 was integrated into the cell line expressing mNG::FCP4/TbKin15 and the flagellum tip marker. Induction of FCP4/TbKin15 RNAi with doxycycline did not cause a growth defect, with the induced cells growing at a similar rate to the non‐induced cells (Figure 4a). The success of the RNAi was confirmed by the absence of mNG::FCP4/TbKin15 signal in the induced populations (Figure S7a). There was a visible depletion of mNG::FCP4/TbKin15 signal at the groove 24 h after induction, with only 11% of cells retaining a mNG::FCP4/TbKin15 signal, and this reduction in signal was seen throughout the 72‐h time course. The depletion of FCP4/TbKin15 had little effect on cell cycle progression with only a minor reduction in the number of 1K1N cells and a slight increase in the proportion of 2K1N and 2K2N cells seen (Figure 4b).

To determine if the depletion of FCP4/TbKin15 caused any defects in flagellum tip attachment, the position of the new flagellum tip along the cell body was analyzed using the flagellum tip marker as a reference in cells with two flagella (Figure 4c). FCP4/TbKin15 RNAi caused a reduction in the number of cells with a new flagellum tip signal present between the kinetoplast and nucleus with an increase in the number of cells with a free new flagellum tip (Figure 4c). Next, we quantified the number of 2K1N cells with a free new flagellum tip (Figure 4d,e). We saw that the depletion of FCP4/TbKin15 caused a large increase in the number of 2K1N cells with a free new flagellum tip (Figure 4d,e). This confirmed that the depletion of FCP4/TbKin15 caused premature new flagellum tip release from the groove. The premature release of the new flagellum tip was supported by SEM, with ~64% of two flagella cells (*n* = 161) having a free new flagellum tip 72 h post‐induction compared to ~23% in the non‐induced cell line (*n* = 271) (Figure S7b). Despite this, ~36% of 2F cells in the FCP4/TbKin15 RNAi cell line still possessed a groove (Figure S7b), suggesting that the depletion of FCP4/TbKin15 did not prevent new flagellum tip embedding.

### 2.6 Localization of FCP4/TbKin15 and FCP7 is independent of each other

Given the premature release of the new flagellum tip with the loss of either FCP4/TbKin15 or FCP7, we investigated whether there was a dependency relationship between them. We generated a double‐tagged cell line in which FCP7 was tagged with mNeonGreen and FCP4/TbKin15 was tagged with mScarlet (Figure 4f). Despite both tagged proteins localizing to the groove, the signals did not overlap and were adjacent with FCP7 generally positioned closer to the cell body, suggesting that FCP7 may be positioned within the cell body domain of the groove. Next, we examined the localization of FCP4/TbKin15 in the FCP7 deletion cell line using the antibody mAb62, which recognizes FCP4/TbKin15 (Abeywickrema et al., 2019). In the parental cell line, mAb62 had a signal consistent with the localization of FCP4/TbKin15 in the groove (Figure 4g). While, in the FCP7 deletion cell line, mAb62 stained the tip of the detached new flagellum, indicating that FCP7 is not required for the localization of FCP4/TbKin15 to the new flagellum tip. We then performed the reciprocal experiment by introducing the FCP4/TbKin15 RNAi plasmid into the cell line expressing FCP7::mNG (Figure 4h). On induction of FCP4/TbKin15 RNAi, we saw no loss of mNG::FCP7 signal. Furthermore, the position of the tip marker was more anterior than the mNG::FCP7 signal, suggesting that the tip was not embedded in the groove. This suggests that the localization of mNG::FCP7 does not require the presence of FCP4/TbKin15.

### 2.7 Presence of groove proteins does not correlate with fully embedded new flagellum tip

As the groove was identified in *T. brucei*, we wanted to determine whether its protein components are present in other kinetoplastids. To investigate this, the presence or absence of all 13 identified groove proteins was determined in a set of parasitic and free‐living kinetoplastid species by reciprocal best BLAST (Figure S8a). FAZ10, FPRC, CIF4, FLAM3, and FCP5/TbULK4 were present in all the kinetoplastids interrogated. FAZ2 was present in all kinetoplastids examined except for *Bodo saltans*, while CIF1 was found in *T. vivax*, *T. congolense*, *L. major*, and *L. pyrrhocoris*. Five groove proteins (FCP7, FAZ16, CIF3, FAZ45, and FAZ15) were further restricted and only present in *T. vivax*, *T. congolense*, and *T. cruzi*. Finally, FCP4/TbKin15 was present in *T. vivax* and *T. congolense* and also in *B. saltans* but not in the other species.

All the identified groove proteins were found in the closely related species *T. congolense* and *T. vivax*. To determine whether the groove structure itself was also present, we examined BSF *T. congolense* and *T. vivax* cells by SBF‐SEM. In *T. brucei* cells with a short new flagellum (3 μm), the new flagellum tip was positioned in a shallow indentation in the cell body membrane (Figure S8b). Slices through the reconstructed area around the new flagellum tip showed that this indentation was not deep, with the new flagellum tip only partially embedded. A comparable model of *T. congolense* with a short new flagellum (4 μm) showed that the new flagellum tip was also positioned in a shallow indentation in the cell body membrane (Figure S8c). Unlike *T. brucei* and *T. congolense*, a model of a *T. vivax* cell with a short new flagellum (4 μm) showed that the new flagellum tip was actually tilting away from the cell body (Figure S8d). Slices through the reconstructed area around the new flagellum tip supported this observation, showing the most distal point of the new flagellum was not associated with the cell body membrane.

**FIGURE 5:**
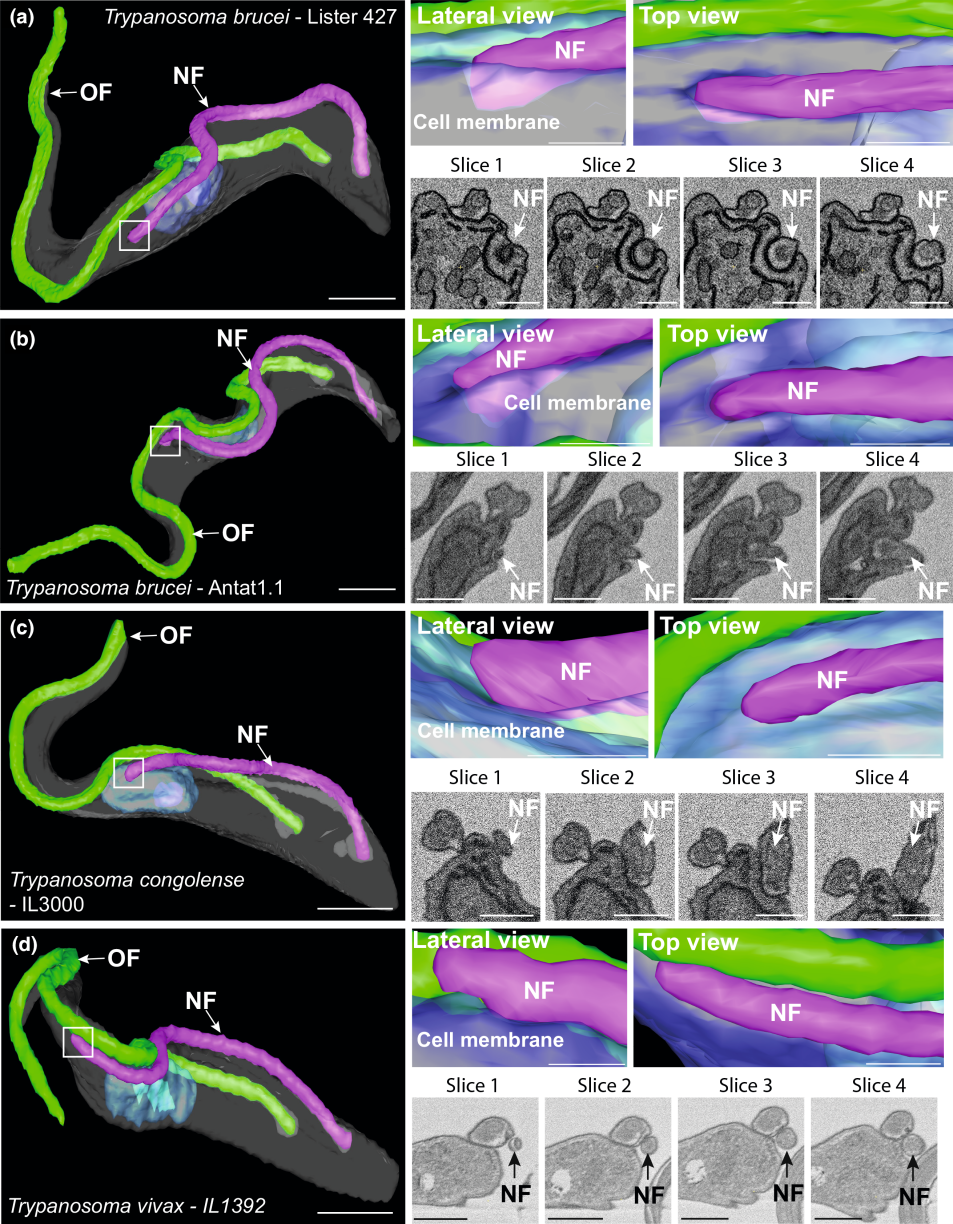
A canonical groove is not present in *Trypanosoma congolense* or *Trypanosoma vivax*. (a) Surface rendering of a *T. brucei* cell (Lister 427 culture strain) with a long new flagellum, with the distal tip fully embedded in a deep groove in the cell body plasma membrane (inset box). SBF‐SEM raw data of the reconstructed region shows distal tip of new flagellum fully embedded in a deep groove in the cell body. Top view of (a) shows that distal tip of new flagellum is positioned away from the old flagellum. (b) Surface rendering of a *T. brucei* cell (Antat1.1 strain) with a long new flagellum, with the distal tip fully embedded in a deep groove in the cell body plasma membrane (inset box), similar to the Lister 427 strain. SBF‐SEM raw data of the reconstructed region shows distal tip of new flagellum fully embedded in a deep groove in the cell body. Top view of (b) shows that distal tip of new flagellum is positioned away from the old flagellum. (c) Surface rendering of a *T. congolense* cell with a long new flagellum with the distal tip positioned in a shallow indentation of the cell body plasma membrane (inset box). SBF‐SEM raw data of the reconstructed region shows distal tip of new flagellum is not fully embedded in the cell body. Top view of (c) shows that distal tip of new flagellum positioned away from the old flagellum. (d) Surface rendering of a *T. vivax* cell with a long new flagellum with the distal tip tilting away from the cell body plasma membrane (inset box). SBF‐SEM raw data of the reconstructed region shows distal tip of new flagellum tilting away from the cell body plasma membrane. Top view of (d) shows that distal tip of new flagellum closely associated with the old flagellum. *N* ≥ 3 models per species. Key: NF = new flagellum; OF = old flagellum. Scale of whole cell models = 2 μm. Scale of enlarged model images and SBF‐SEM raw data slices = 500 nm.

In cells with a longer new flagellum, clear differences between the three species were evident (Figure 5). In *T. brucei* cells (Lister 427 culture strain) with a longer new flagellum (14.8 μm), the new flagellum tip was fully embedded in a deep groove in the cell body membrane (Figure 5a), representing the canonical groove structure observed in previous studies (Hughes et al., 2013). Slices through the reconstructed area around the new flagellum tip supported this, showing that the new flagellum tip was fully embedded. Furthermore, at this length, the new flagellum tip was positioned away from the side of the old flagellum, suggesting that as the new flagellum elongated, the tip moved further away from the old flagellum, possibly due to the insertion of microtubules between the two flagella. We confirmed the groove structural arrangement for *T. brucei* by examining an additional strain of *T. brucei*. We examined the pleomorphic *T. brucei* Antat1.1 strain and found that these parasites also possess a new flagellum with the tip fully embedded in the groove when the new flagellum measures 14.6 μm (Figure 5b).

Contrary to both *T. brucei* strains, the new flagellum tip of *T. congolense* remained positioned in a shallow indentation in the cell body membrane, and the depth remaining unchanged from when the new flagellum was short (Figure 5c). Similar to *T. brucei*, the new flagellum was positioned further away from the side of the old flagellum at this length. *T. vivax* differed from *T. brucei* or *T. congolense*, with the new flagellum tip of *T. vivax* still tilted away from the cell body when the new flagellum was longer (14.8 μm) (Figure 5d). Slices through the reconstructed area around the new flagellum tip supported this, showing that the most distal point of the new flagellum was not associated with the cell body. Interestingly, the tip of the new flagellum remained touching the old flagellum, as seen in the FC of the *T. brucei* PCF (Briggs et al., 2004; Moreira‐Leite et al., 2001). These data demonstrate that despite the protein conservation between these three closely related African trypanosomatid parasites, each species displays a different structural organization between the new flagellum tip, cell body, and old flagellum.

## 3 DISCUSSION

The groove is a mobile transmembrane junction connecting the tip of the new flagellum to the underlying cytoskeleton. Here, we identified 13 proteins that localized to the groove, with five distinct localization patterns observed ranging from groove specific to multiple cellular locations including the groove. The functional analysis of a subset of these proteins revealed that the groove is composed of proteins with distinct roles in either new flagellum tip attachment and/or cytokinesis.

The deletion of CIF4 and FCP7 and the RNAi depletion of FCP4/TbKin15 resulted in the premature release of the new flagellum tip from the groove, suggesting that these proteins have an important role in the attachment of the new flagellum to the groove junction. Further to this, the groove localization of FCP4/TbKin15 and FCP7 was lost before or around the time of groove resolution. FCP4/TbKin15 and FCP7 specifically function in maintaining tip attachment (as their depletion and deletion of both had a limited effect on the cell cycle), whereas deletion of CIF4 affected the cell cycle in addition to tip attachment. The cytokinesis defect observed after CIF4 deletion was expected given the role of this protein in cytokinesis in PCFs (Hu et al., 2019) and its association with CIF protein complex, which is required for cytokinesis in both PCFs and BSFs (Hilton et al., 2018; Kurasawa et al., 2018; McAllaster et al., 2015; Sinclair‐Davis et al., 2017; Zhou et al., 2016; Zhou et al., 2018). It is unlikely that a cytokinesis defect would impact directly on groove resolution as this occurs before cytokinesis. Moreover, deletion of FAZ15 and FAZ16 demonstrated that a defect in cytokinesis was possible without affecting tip release. Premature tip release after CIF4 deletion may therefore be due to a general destabilization of the groove structure. FAZ15 and FAZ16 have been identified as interactors of TbPLK at the tip of the new FAZ in PCFs (McAllaster et al., 2015). The deletion of both proteins in our study caused cytokinesis defects, resulting in an accumulation of multiflagellated and multinucleic cells, compatible with a role in cytokinesis. TbPLK phosphorylates TOEFAZ1/CIF1, which is a core component of the CIF complex that is required for cytokinesis in both PCFs and BSFs (McAllaster et al., 2015; Sinclair‐Davis et al., 2017; Zhou et al., 2016). FAZ15 and FAZ16 as interactors of TbPLK may therefore form part of the protein complex required for cytokinesis in BSFs along with CIF4. The groove, likely due to its association with the distal FAZ of the new flagellum, may be involved in the transport of proteins involved in the cytokinetic complex to the site of cleavage furrow ingression towards the anterior of the cell.

In PCFs, FCP4/TbKin15, a member of the kinesin‐15 family of microtubule plus‐end‐directed motors, is responsible for linking the plus ends of the extending new flagellum axoneme microtubules to the flagella connector membrane junction (Varga et al., 2017). Here, FCP4/TbKin15 localized specifically to the flagellar groove as a distinct foci around the new flagellum tip marker, suggesting that FCP4/TbKin15 is localized to the flagellum side of the groove cell junction. As FCP4/TbKin15 links the axonemal microtubules to the flagella connector in the PCF (Varga et al., 2017), this function may be conserved in the BSF, with FCP4/TbKin15 potentially linking the axonemal microtubules of the new flagellum to the groove junction during new flagellum growth. Moreover, depletion of FCP4/TbKin15 in PCFs led to premature release of the new flagellum tip, mirroring the phenotype we observed in BSFs. From our imaging, FCP7 appears to be localized to the cell body side of the groove and may form part of the connections in this part of the structure. Together, our findings suggest that the tethering of the new flagellum tip in the groove involves a complex arrangement of several proteins from both the flagellum and cell body side of the junction. In contrast to the premature release of the tip, the deletion of FAZ45 appeared to cause delayed new flagellum tip release from the groove, which appeared to have an impact on cytokinesis as there was a drop in 1K1N cells. This suggests that groove resolution is a complex process with a number of regulatory steps and the presence of FAZ45 is required to initiate groove resolution; without this protein, the tip is not released in a timely manner.

There is limited overlap in composition between the groove and flagella connector as previous transcriptomic and proteomic analysis suggested, with significant life cycle innovation in each of these structures (Siegel et al., 2010; Varga et al., 2017). However, there is a core conserved set of components found in both the groove and flagella connector, including FCP4/TbKin15, FCP5/TbULK4, and FCP7, with the role of FCP4/TbKin15 and FCP7 conserved between the different life cycle stages. In the BSF, FCP5/TbULK4 also localizes elsewhere in the cell, suggesting that FCP5/TbULK4 has additional roles beyond new flagellum tip attachment. Our discovery of the identified groove proteins has an obvious caveat; in that it was founded on the PCF localization database, TrypTag. Since BSFs and PCFs are biologically and morphogenetically different (Wheeler et al., 2013), this creates the potential for missing BSF‐specific proteins during our initial candidate search, and there may be key groove proteins absent from our screen.

In part, the groove is an elaboration of the FAZ, with an electron‐dense network of filaments underlying its cytoplasmic face and filaments radiating across the cell body plasma membrane through the flagellum membrane (Hughes et al., 2013). Our results confirm this, as we localized several known FAZ proteins to the groove—FLAM3, FAZ2, FAZ10, FAZ15, and FAZ16 (An & Li, 2018; Moreira et al., 2017; Rotureau et al., 2014; Sunter & Gull, 2016; Sunter, Varga, et al., 2015). The groove, therefore, contains FAZ proteins from both the flagellum membrane and cell body membrane regions of the FAZ that are important for FAZ structure and stability (Sunter, Benz, et al., 2015). Despite the presence of several FAZ proteins in the groove, the filament extensions in the groove junction are decidedly more complex than the canonical FAZ filament as these extensions have to undergo dynamic change to facilitate the release of the new flagellum tip during groove resolution.

We were able to determine that groove resolution occurs post‐mitosis, before cytokinesis initiates, yet the positioning of the cytokinesis furrow and formation of the new anterior cell tip still needs to be defined. Furthermore, post‐mitosis, the depth of the groove decreased as it approached the anterior cell tip, likely as part of the groove resolution process. We observed that at around this point, the new flagellum length surpassed the old flagellum length. In the reconstructed 2K2N cells in which the groove had resolved, the average length of the new flagellum was longer (1.05×) than the average length of the old flagellum. By the start of cytokinesis, the length of the new flagellum now matches that of the old flagellum, suggesting a short period of flagellum disassembly before the flagellum is “locked” at its mature length (Bertiaux et al., 2018). This contrasts with the PCF in which cytokinesis occurs with the new flagellum shorter than the old flagellum, with new flagellum assembly continuing in the daughter cell inheriting the new flagellum (Calvo‐Álvarez et al., 2021; Farr & Gull, 2009).

The groove was hypothesized to be specifically adapted to reduce the exposure of parasite proteins present in an external structure, like the flagella connector, to the mammalian immune system (Hughes et al., 2013). We examined the growing new flagellum tip in the closely related species *T. vivax* and *T. congolense* and found that despite all 13 identified groove proteins being present in these organisms, they did not possess a canonical groove with the flagellum tip embedded within the cell body. In *T. congolense*, we observed that the tip of the new flagellum was positioned in a shallow indentation in the cell body plasma membrane but was never fully embedded. In *T. vivax*, we saw that the new flagellum tip was not associated with the cell body membrane in the same way as *T. brucei* or *T. congolense*. Instead, the new flagellum tip was tilted away from the cell body and was in close proximity to the lateral surface of the old flagellum, reminiscent of the *T. brucei* flagella connector. Moreover, *T. vivax* possesses orthologs of all the flagella connector proteins, suggesting the possibility that BSF *T. vivax* utilizes this transmembrane junction to tether the new flagellum tip. Our analysis suggests that the deep groove only began to evolve after the speciation of *T. vivax*, and therefore, despite the close phylogenetic relationship between these three trypanosomatids, all three display species‐specific adaptations at the tip of the growing new flagellum in the BSF.

The embedded flagellum tip was only observed in *T. brucei*, and this now seems unlikely to be an adaptation to reduce exposure of an invariant structure to the immune system of the mammalian host. However, in their mammalian hosts, *T. brucei*, *T. vivax*, and *T. congolense* have a level of tissue tropism, with *T. brucei* considered a tissue parasite, whereas *T. congolense* and *T. vivax* generally remain in the vasculature, though there is evidence for them both to invade tissues (reviewed in Pereira et al., 2019). The different environments these parasites colonize within the host may generate different immune pressures that might be selected for the groove in *T. brucei*. In addition, these three species have distinct patterns of movement and velocity in blood and solutions of different viscosities, and these different structures at the tip of the new flagellum may enable these different styles of motility while the parasite is assembling a new flagellum alongside the old one (Bargul et al., 2016).

Here, we have identified a cohort of groove proteins, with a number of these involved in the maintenance of this mobile transmembrane junction. However, despite the presence of these groove proteins in closely related African trypanosome species, there is limited structural conservation between the structures associated with the growing new flagellum tip. This points to the rapid evolution of cytoskeletal‐membrane structures without the need for large changes in gene complement, especially those exposed to the host immune system, and this likely reflects the niche specialization of these parasites.

## 4 MATERIALS AND METHODS

### 4.1 Groove candidate identification

The TrypTag database (http://tryptag.org/) (Dean et al., 2017) was mined for proteins localizing to the (i) distal FAZ; (ii) anterior cell tip; (iii) flagella connector. Forty‐five proteins were chosen for a localization screen. Groove proteins were examined by reciprocal best BLAST. We interrogated the genomes of *Trypanosoma vivax* (Y486), *Trypanosoma congolense* (IL3000), *Trypanosoma cruzi* (Dmc28 2018), *Leishmania major* (Friedlin), *Blechomonas ayalai* (B08‐376), *Leptomonas pyrrhocoris* (H10), *Paratrypanosoma confusum* (CUL13), and *Bodo saltans* (Lake Konstanz) for orthologous proteins with an e‐value cut‐off of e^−10^.

### 4.2 Cell culture

*T. brucei* bloodstream Lister 4,27 1339 cells were grown in HMI‐9 (Gibco, UK) medium supplemented with heat‐inactivated 15% FCS (Gibco, UK), at 37°C in a 5% CO_2_ incubator. Cells were harvested during log phase (6 × 10^5^ – 1 × 10^6^ cells/ml). For growth curves, cells were counted every 24 h using an automated cell counter and split to 1 × 10^5^ cells/ml. Bloodstream form *T. congolense* cells (IL3000 strain) were grown and maintained at 37°C in a 5% CO_2_ incubator in basal medium supplemented with complete Tc‐BSF3 medium. Basal medium consists of MEM supplemented with 50 mM acid HEPES, 52 mM NaHCO_3_, 11 mM D‐Glucose, 2 mM sodium pyruvate, 0.08 mM adenosine, 0.2 mM hypoxanthine, 0.03 mM thymidine, and 0.05 mM bathocuproine sulfate acid. Basal medium pH was adjusted to 7.3 and filtered. To complete the medium, basal medium was supplemented with 15% FCS (Gibco, UK), 5% Serum Plus™ II Medium Supplement (Sigma, UK), 1.4% β‐mercaptoethanol, and 200 mM glutamine. Cells were harvested during log phase (5 × 10^5^ – 6 × 10^6^ cells/ml).

### 4.3 Harvesting *T. vivax* bloodstream form cells

Bloodstream form *T. vivax* (IL1392 strain) was derived from the Y486 strain and was kindly provided by Dr Delphine Autheman and Professor Gavin Wright (University of York). The parasites were maintained by weekly serial passage in wild‐type female BALB/c mice according to Autheman et al. (2021). Fifteen mice were infected and harvested 6 days post‐infection to acquire parasites in log phase. Parasites were collected from the blood via intracardiac puncture using heparin before diluting the blood with 20 mM of PBS‐glucose solution. Blood was centrifuged to separate parasites and red blood cells. The supernatant containing the parasites was removed and centrifuged. The parasite solution was resuspended in 20 mM PBS‐glucose. The *T. vivax* sample was sent to Oxford Brookes University in 2.5% EM‐grade glutaraldehyde in 0.1 M phosphate buffer solution (pH 7.4) and stored at 4°C.

### 4.4 Cell line generation

The BSF 427 1339 cell line contains an integrated construct that encodes the T7 RNA polymerase, TetR, and Cas9 nuclease (Beneke et al., 2017). Primers for template and guide PCR were designed using the primer design tool in http://www.leishgedit.net/Home.html. Constructs for endogenous tagging and gene deletion were generated by long‐primer PCR and transfection of cells, as described by Dean et al. (2015) and Beneke et al. (2017). Constructs were designed to tag either the N or C terminal of the gene of interest based on their respective localization pattern seen on TrypTag. Template plasmids pPOTv6 – mScarlet – blast, pPOTv6 – mNeonGreen – hygromycin and pPOTv6 – mNeonGreen – G418 were used. The PCR products were combined with 3 × 10^7^ cells/ml and transfected using the Amaxa Nucleofactor II system. Transfectants were selected with 5 μg/ml blasticidin, 2.5 μg/ml G418 (neomycin), 5 μg/ml hygromycin, and 0.1 μg/ml puromycin as appropriate. To confirm the successful deletion of the gene of interest, genomic DNA was extracted using the DNeasy Blood and Tissue kit. Primers were designed to amplify the ORF of the gene of interest. PCR amplification was checked using gel electrophoresis using parental gDNA and ddH_2_O as positive and negative controls. An RNAi plasmid against FCP4/TbKin15 (Hairpin pDEX777 #9) was kindly provided by Dr Vladimir Varga (Institute of Molecular Genetics of the Czech Academy of Sciences, Prague). The plasmid was linearized with NotI prior to transfection into the FCP4/TbKin15 cell line endogenously tagged with mNG and was selected for with 5 μg/ml phleomycin. Doxycycline (1 μg/ml) was used to induce FCP4/TbKin15 RNAi.

### 4.5 Fluorescence light microscopy

For imaging of native fluorescence, *T. brucei* cells were harvested from culture in log phase and centrifuged (8 min, 800 g). The cells were resuspended in 1 ml 1X Dulbecco's Modified Eagle Medium (DMEM) supplemented with 0.001% Hoechst‐33342, to label the DNA, and centrifuged again, before being washed in 1 ml DMEM only. The cells were resuspended in 20 μl DMEM. 20 μl of 0.04% methanol‐free formaldehyde (16% stock; TAAB) was added before mounting 2.4 μl of cells onto glass slides. For immunofluorescence, *T. brucei* cells were harvested from culture in log phase, centrifuged (8 min, 800 g), and washed twice with 1 ml DMEM. Cells were resuspended in 0.5 ml DMEM and fixed by the addition of 0.5 ml 2% formaldehyde in vPBS for 5 min. Cells were permeabilized with 0.1% NP‐40 for 5 min before adding 10 ml DMEM and being centrifuged for 8 min at 800 g. Cells were resuspended in 100 μl DMEM and settled onto poly‐L‐lysine slides for 10 min. Slides were blocked with PBS and 1% BSA for 1 h at room temperature. The primary antibody, mAb62 (1:100) in 1% BSA, was added to the slides for 1 h at room temperature before washing 4 × 5 min in PBS. Next, goat anti‐mouse secondary antibody (1:200) conjugated to Alexa Fluor‐488 was added to the slides for 45 min at room temperature before washing 4 × 5 min in PBS. Slides were mounted using 90% glycerol buffered with phosphate buffer at pH 7.4. The cells were imaged on a Zeiss Imager‐Z2 microscope using the Zen Blue software and a 63× oil‐immersion objective. Images were processed using ImageJ with an automated script adapted from Wheeler (2020).

### 4.6 Scanning electron microscopy

Cells were harvested in log phase and 2.5% EM‐grade glutaraldehyde (TAAB) was added directly to the culture flask. Cells were centrifuged (8 min, 800 g) before being resuspended in buffered fixative containing 2.5% glutaraldehyde and 0.1 M phosphate buffer solution. The cells were washed again in buffered fixative, resuspending thoroughly before incubation—1 h at room temperature. The cells were settled onto 13 mm round glass coverslips coated with 0.1% poly‐L‐lysine, washed with ddH_2_O, and taken through an ethanol dehydration series, each step lasting 5 min: 30%, 50%, 70%, and 90% in ddH_2_O followed by three incubations in 100% absolute ethanol. The coverslips were dried using an Autosamdri‐931.GL critical point dryer (Tousimis, Canada) before being coated in 11–13 nm thickness of gold using an Automatic Gold Sputter Coater (Agar Scientific, UK). Samples were imaged using a Hitachi S‐3400 N Scanning Electron Microscope (conditions: 5 Kv, 5–7 mm working distance, 20–50 μA, 260 s image capture).

### 4.7 Serial block‐face scanning electron microscopy

*T. brucei* and *T. congolense* BSF cells were harvested in log phase and fixed directly in culture with 2.5% glutaraldehyde. Cells were centrifuged (8 min, 800 g) and resuspended in fresh buffered fixative and left to incubate for an hour at room temperature. *T. vivax* cells suspended in buffered fixative, as well as the *T. brucei* and *T. congolense* cells, were washed five times (3 min) in 0.1 M phosphate buffer and pelleted by centrifugation (1 min, 14,000 rpm) between each wash. Cells were incubated in 1% osmium tetroxide and 1.5% filtered potassium ferricyanide for 1 h at 4°C in the dark. *T. vivax* cells were washed five times in ddH_2_O, whereas the *T. brucei* and *T. congolense* cells were washed in 0.1 M phosphate buffer five times (3 min per wash), pelleted by centrifugation between washes (1 min, 14,000 rpm). *T. vivax* cells were incubated separately in fresh 1% filtered thiocarbohydrazide for 20 min at room temperature in the dark. The *T. congolense* and *T. brucei* cells were incubated in 1% filtered tannic acid for 30 min at room temperature. Cells were washed three times (5 min) in ddH_2_O before incubating in 2% osmium tetroxide for 30 min at 4°C in the dark. Cells were washed as before and incubated in filtered 0.5–1% uranyl acetate overnight at 4°C in the dark. Cells were washed three times in ddH_2_O (5 min) and then dehydrated by a series of 10 min incubations in 30%, 50%, 70%, 90% ethanol and three changes of 100% absolute ethanol. Cells were incubated in a series of ratios of ethanol to 25% epoxy resin (TAAB 812 hard resin) as follows: 3:1 ethanol/resin (1 h), 1:1 ethanol/resin (2 h), 1:3 ethanol/resin (1 h), then 2–3 changes of 100% resin (6 h each incubation). Resin‐infiltrated pellets were embedded with fresh 100% resin and polymerized at 60°C for 24 h. The sample was trimmed and mounted on an aluminium pin for 3View SBF‐SEM using epoxy conductive resin from Circuitworks™. Resin was polymerized at 60°C for 24 h. Samples were inserted into a Zeiss Merlin scanning electron microscope with Gatan 3View automated sectioning and image capture system. Samples were sectioned using a 3 mm intra‐microscope diamond knife, with a slice thickness of 100 nm. The block‐face was scanned by an electron beam at an accelerating voltage of 1.8 Kv with nitrogen gas administered using a Focal Charge Compensation device. Other conditions include pixel size between 3–5 nm, pixel time of 3–4 μs, and 20 μm aperture. Data sets ranged from 300–1200 sections.

### 4.8 Data processing and model rendering

SBF‐SEM data were processed using Cygwin™ terminal and 3Dmod (IMOD™, University of Colorado; https://bio3d.colorado.edu/imod/). Separate image files were converted from .dm4 format and combined into a single .mrc file before adjusting the voxel size in Z. The data were binned once (reducing file size by combining adjacent pixels), and the data stack flipped and smoothed (averaging of pixels with their neighbors). The data stack was automatically aligned and trimmed. Regions of interest were identified, highlighted, and combined into a single model file before polishing.

### 4.9 Statistical analysis

Statistical analysis was performed using GraphPad Prism version 9.0. A Two‐way ANOVA with Šidák's multiple comparison test was used to determine which of three or more means differed significantly from one another in an analysis of variance. An unpaired Student's *t* test was used to compare the cell cycle and position analysis of the mutant cell lines against the parental cell line.

## Supporting information


Figure S1

## Data Availability

The data that support the findings of this study are available from the corresponding author upon reasonable request.
