# Supplementary material for: Diversity in new flagellum tip attachment in bloodstream form African trypanosomes
Source: Mol Microbiol. 2022 Sep 14;118(5):510–25. doi: 10.1111/mmi.14979 (PMC9826329; doi:10.1111/mmi.14979)
Supplement: Supplementary file 1 — Figure S1 [file MMI-118-510-s001.pdf]

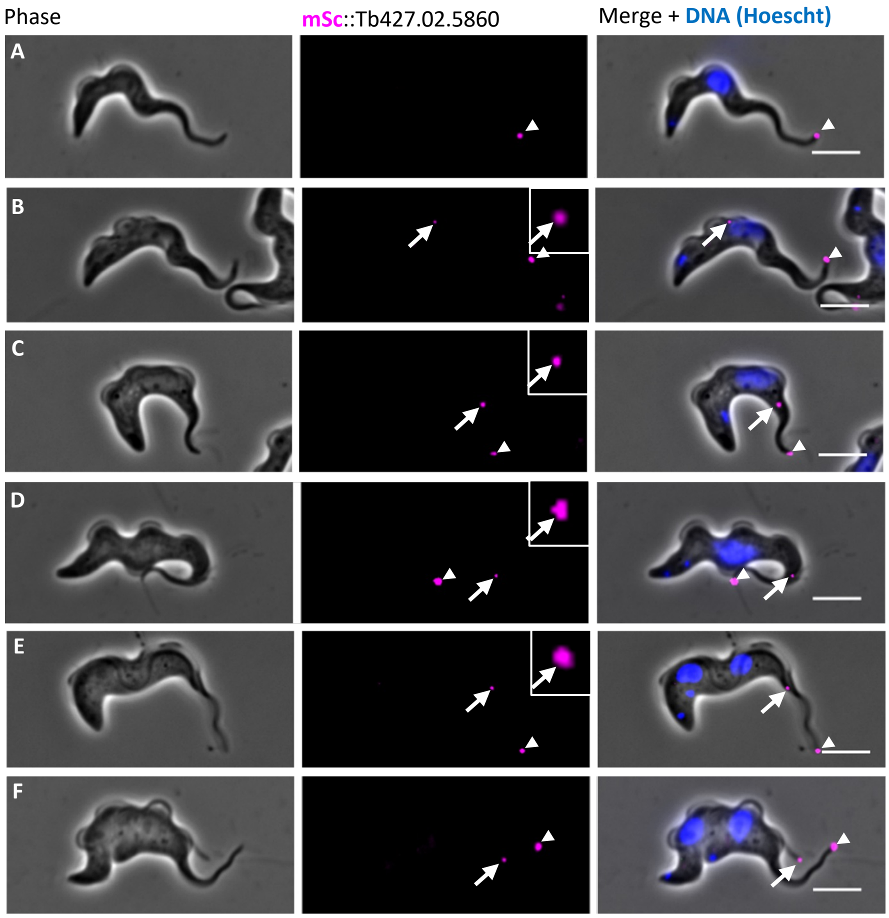

<u>Supplementary figure 1:</u> Tb427 cell line tagged with flagellum tip marker mSc::Tb427.02.5860. A) G1 cell with one kinetoplast and one nucleus, single flagellum tip indicated (magenta, arrowhead); B-D) S-phase cells with divided kinetoplast showing that the new flagellum has migrated from the posterior end of the cell towards the anterior end, both the old flagellum tip (magenta, arrowhead) and new flagellum tip (magenta, arrow, inset) indicated; E) Post-mitotic cell with divided nucleus showing that the new flagellum distal tip (magenta, arrow, inset) is approaching the anterior cell tip; F) During cytokinesis, the new flagellum distal tip is released from the cell body and cytokinesis is initiated to separate the two cells. Old flagellum tip indicated by white arrowhead; new flagellum tip indicated with arrow and inset box. Scale = 5 µm.

<u>Groove only category</u>

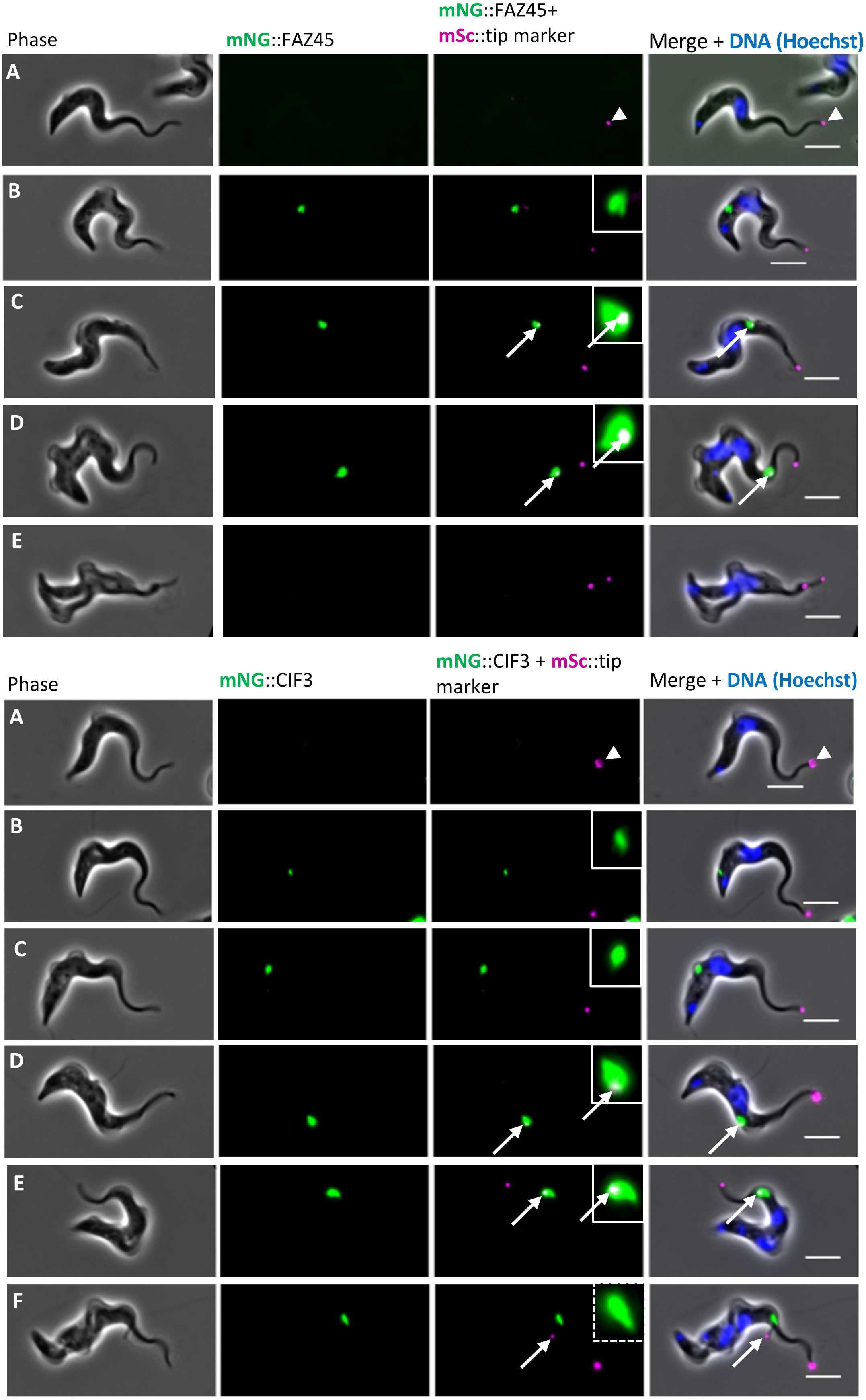

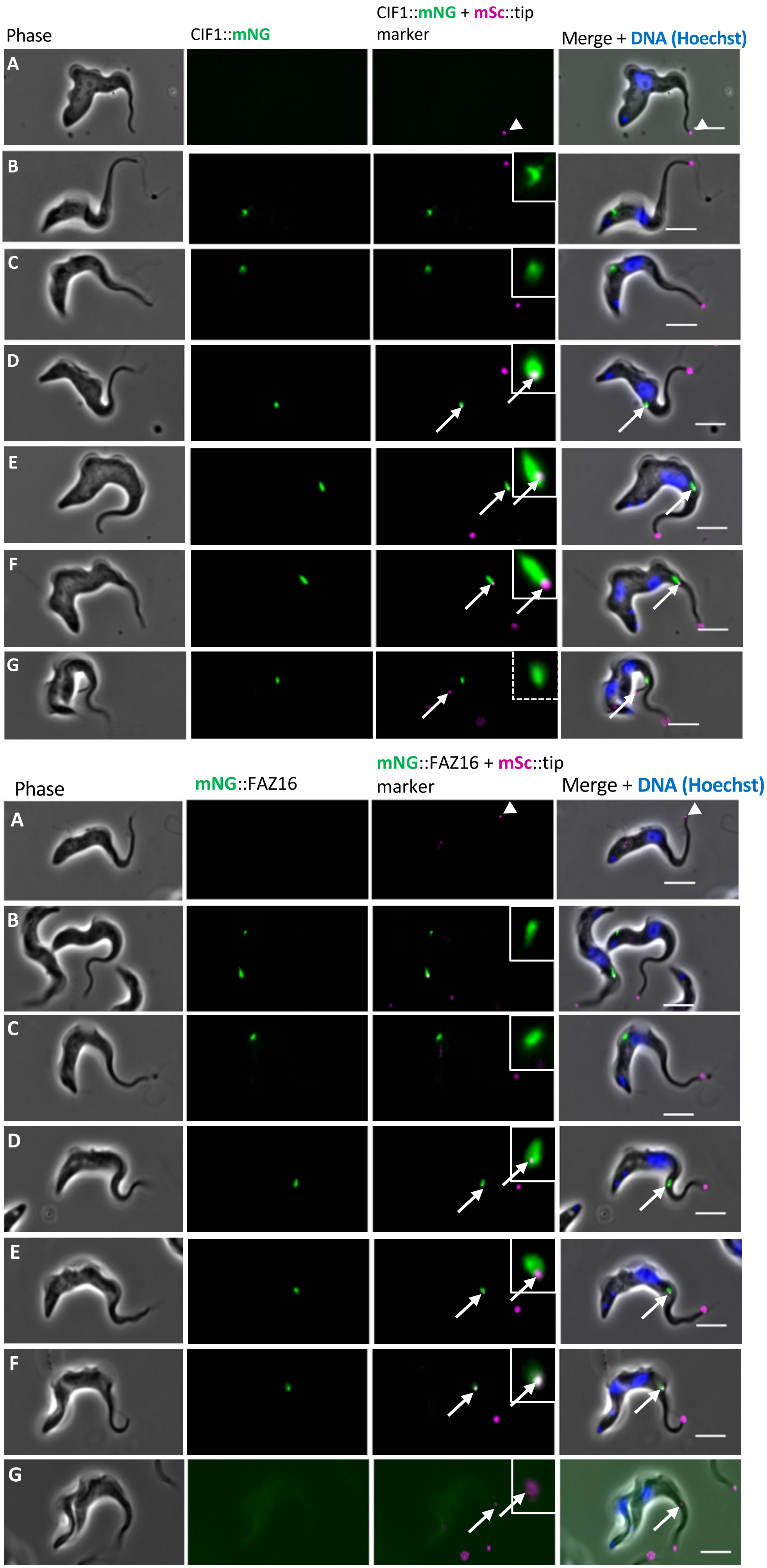

Phase
CIF1::mNG
CIF1::mNG + mSc::tip marker
Merge + DNA (Hoechst)
A
B
C
D
E
F
G
Phase
mNG::FAZ16
mNG::FAZ16 + mSc::tip marker
Merge + DNA (Hoechst)
A
B
C
D
E
F
G

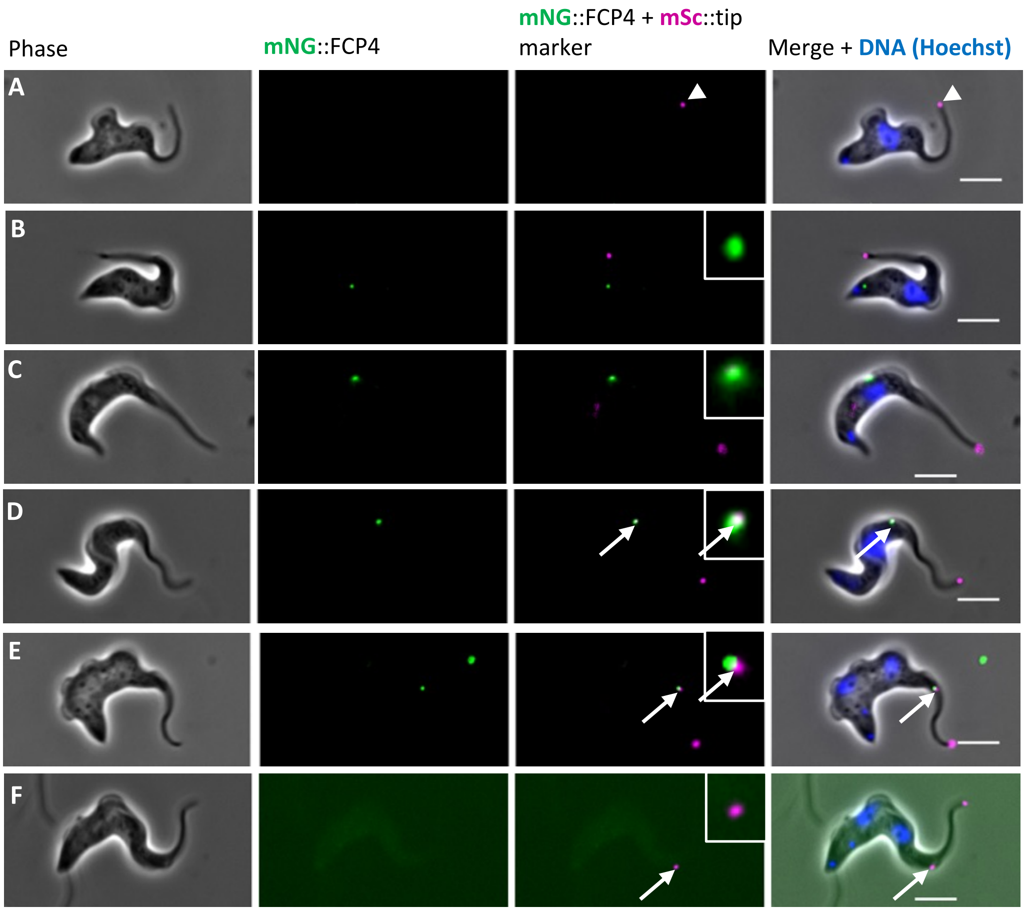
Phase
mNG::FCP4
mNG::FCP4 + mSc::tip marker
Merge + DNA (Hoechst)
A
B
C
D
E
F

Full length FAZ + groove category

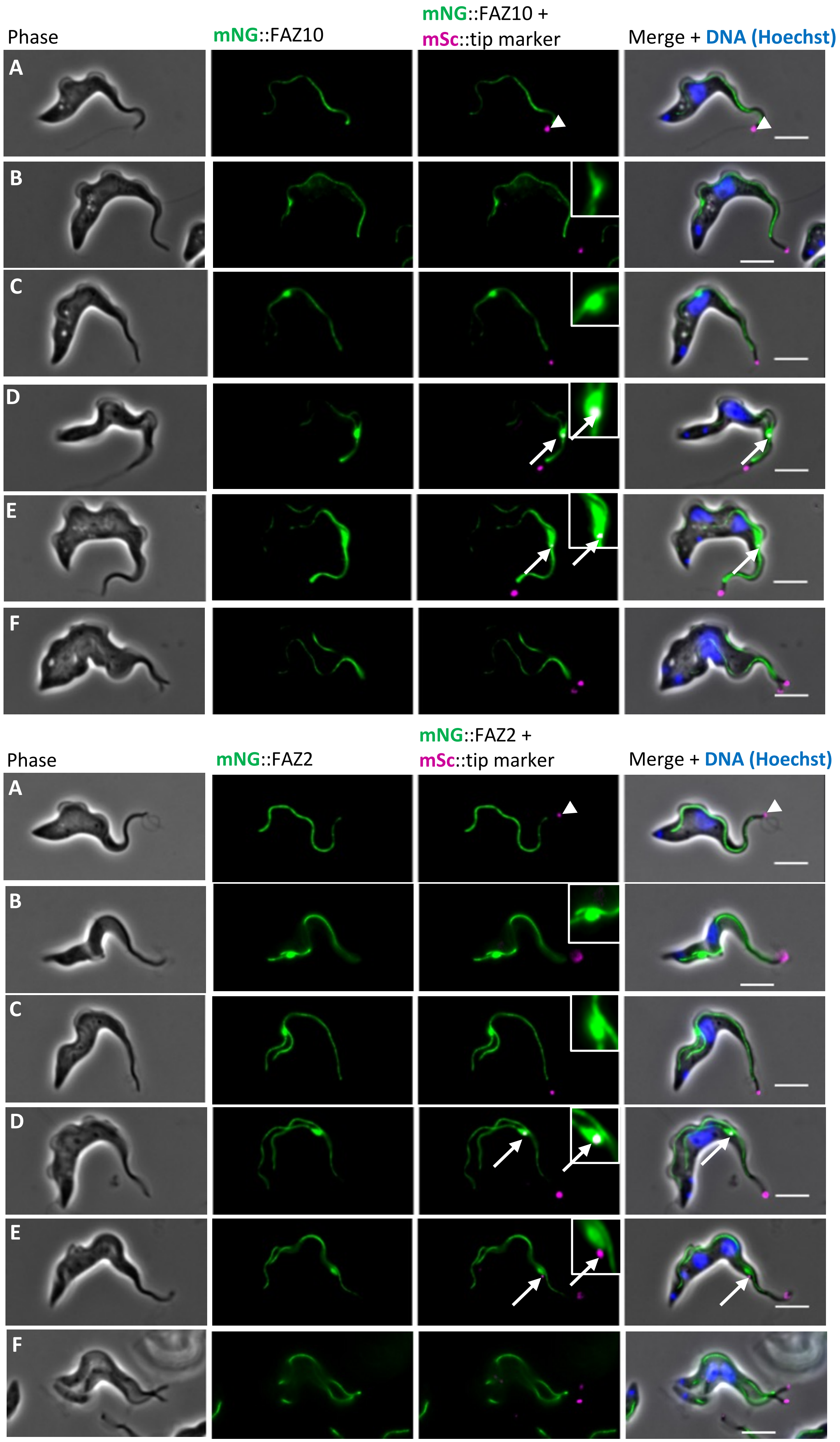

Distal FAZ + groove category

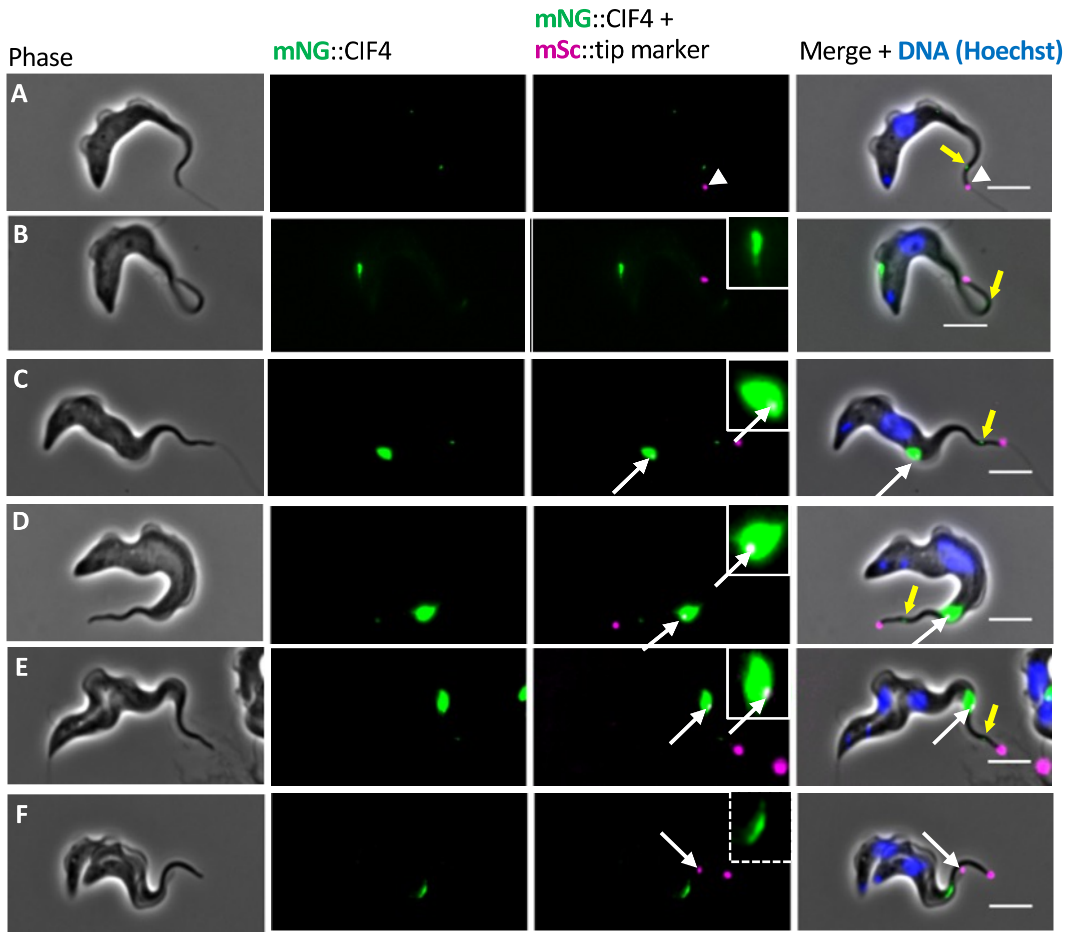

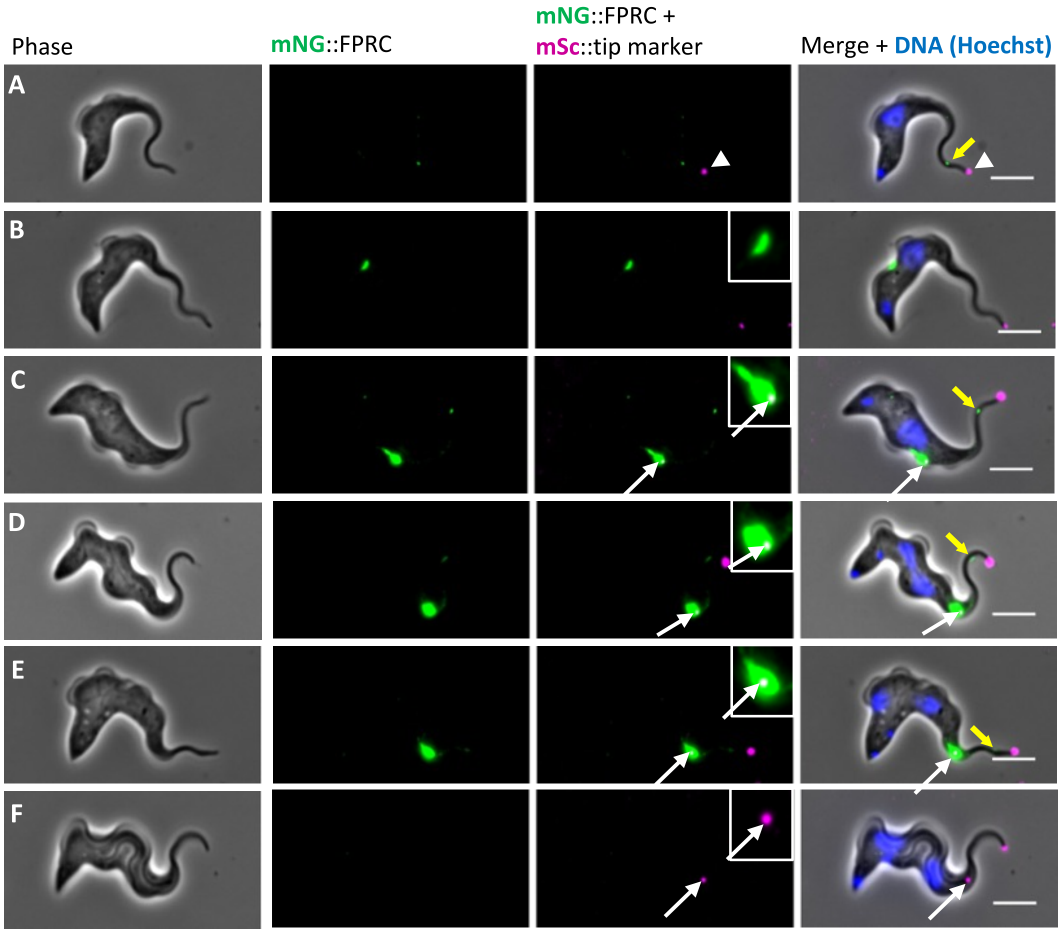

"Comet-tail" category

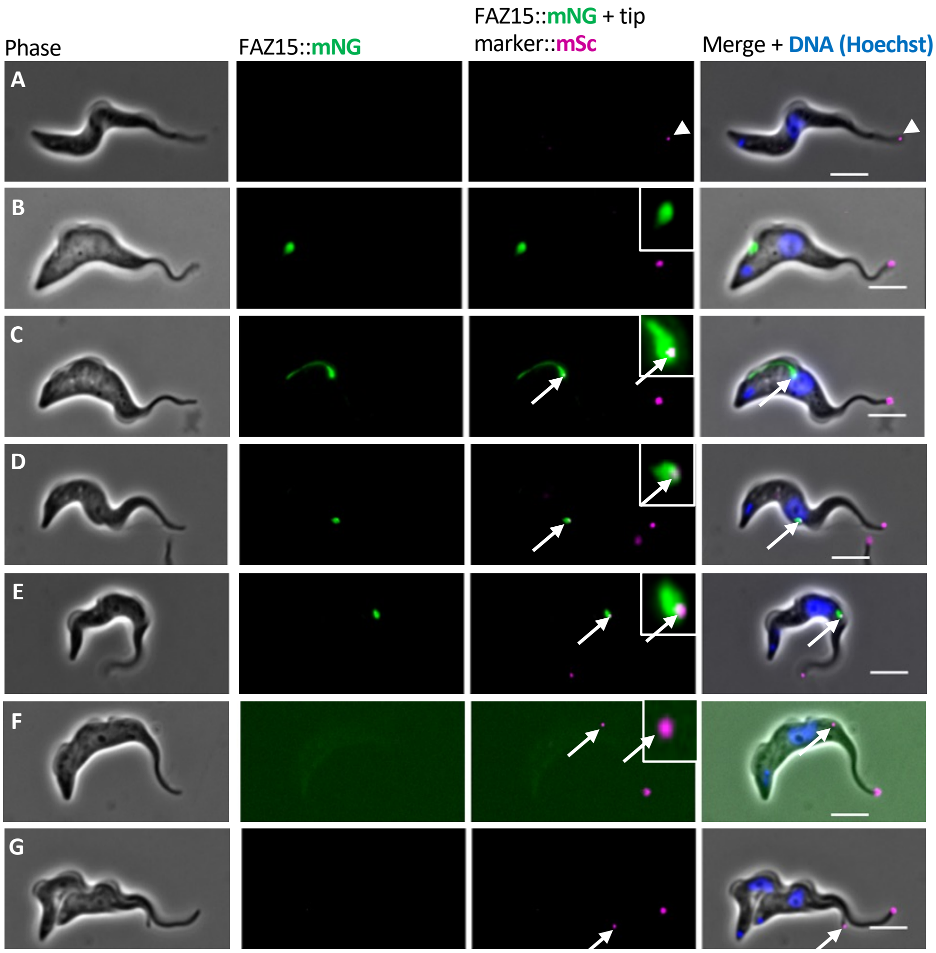

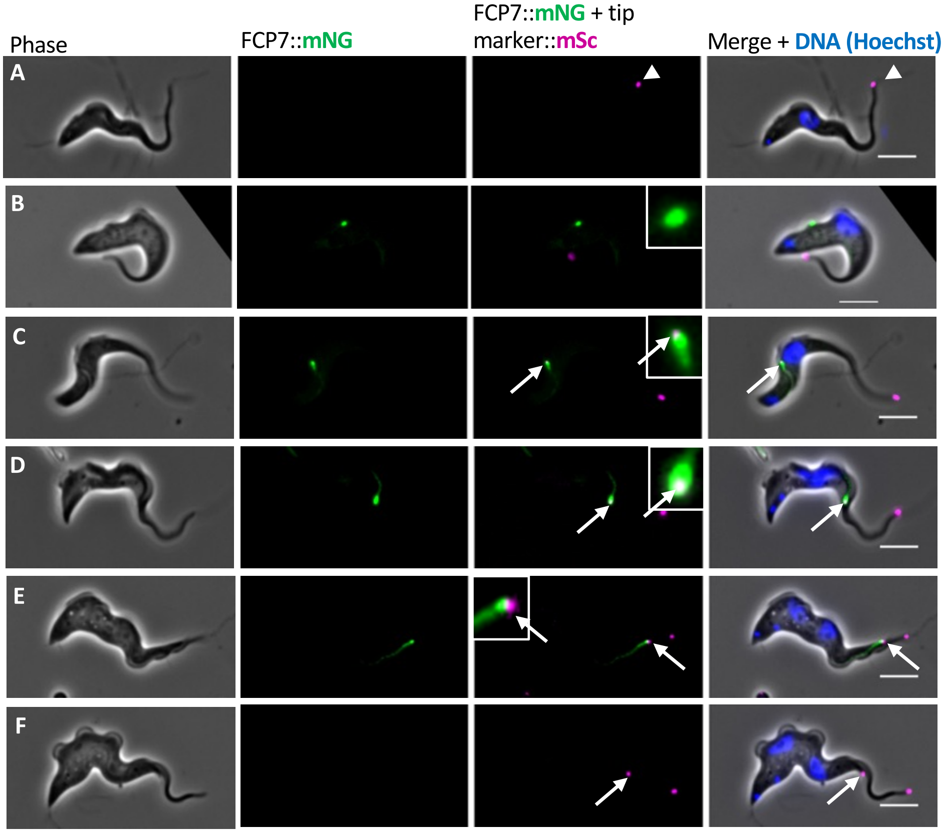

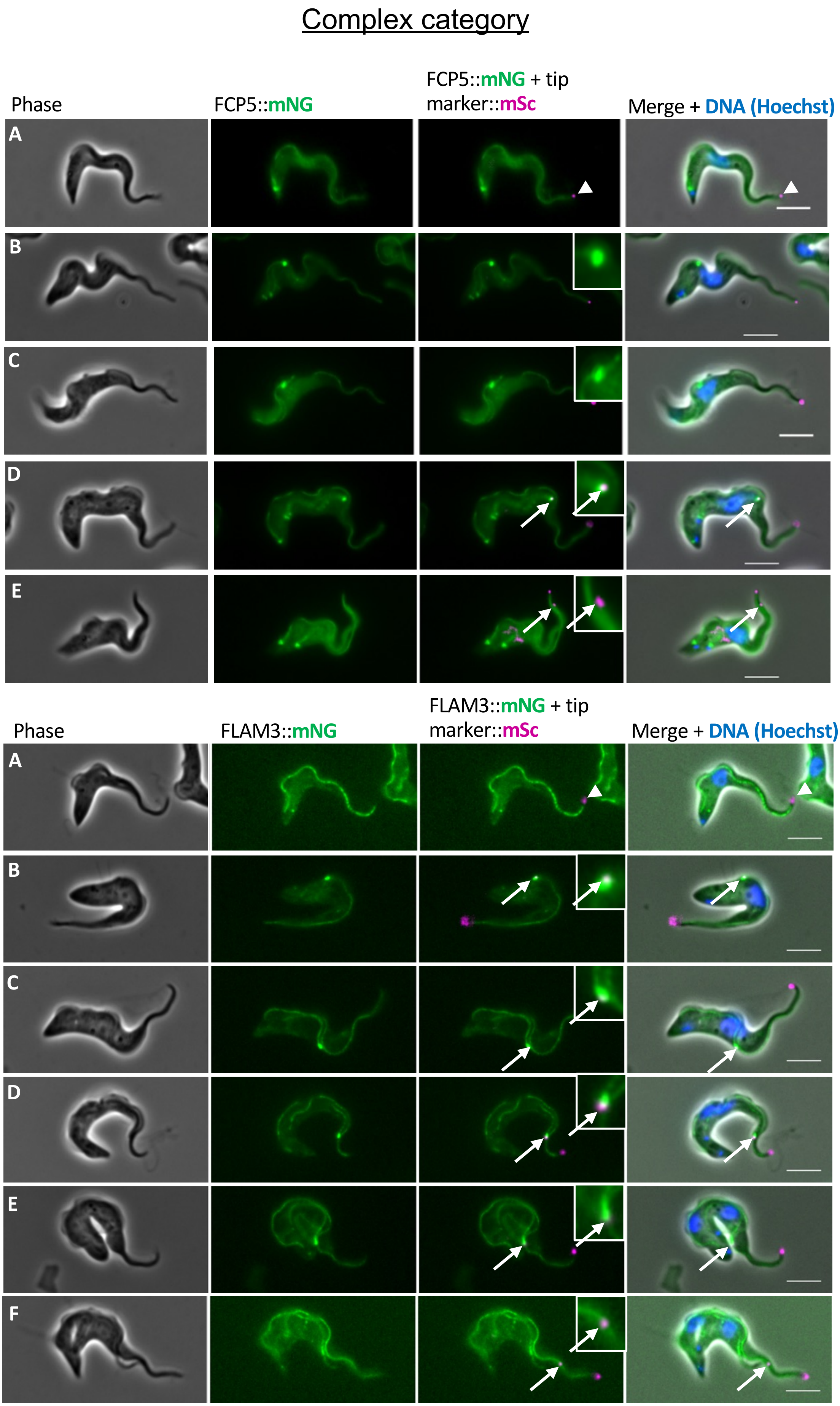
Complex category
Phase
FCP5::mNG
FCP5::mNG + tip marker::mSc
Merge + DNA (Hoechst)
A
B
C
D
E
Phase
FLAM3::mNG
FLAM3::mNG + tip marker::mSc
Merge + DNA (Hoechst)
A
B
C
D
E
F

__Supplementary figure 2:__ **Cell lines expressing mSc::Tb427.02.5860 and the candidate groove protein in mNeonGreen.** Endogenous tagging of 13 groove candidate proteins with mNeonGreen revealed a distinct green elaboration around the distal tip of the new flagellum in BSF cells in each cell line. Scale = 5 µm.

Gene ID	Representative 2K1N cell	Localisation in the BSF
Tb427.04.3740 – FAZ1	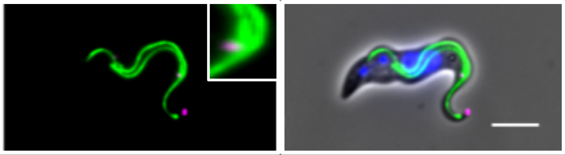	Full length FAZ
Tb427.03.1020 – FAZ13	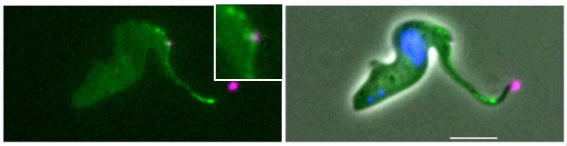	Distal FAZ, anterior cell tip, cytoplasm
Tb427.07.5240 – FAZ21	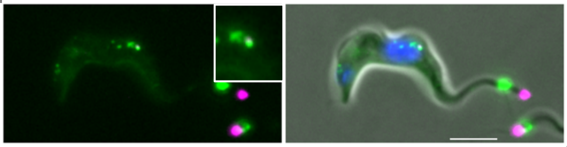	Anterior cell tip, cytoplasm
Tb427.10.840 – FAZ6	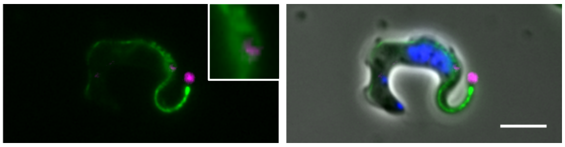	Distal FAZ, anterior cell tip, cytoplasm
Tb427.10.9720 – REAP1	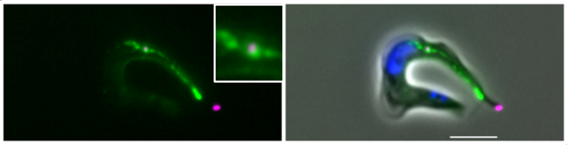	Distal FAZ, anterior cell tip, cytoplasm
Tb427.10.11650	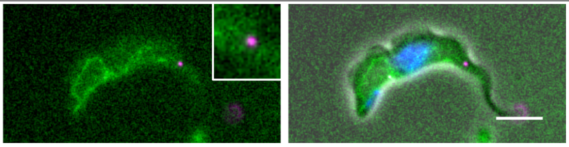	Cytoplasm, endoplasmic reticulum
Tb427tmp.02.0190 – FAZ12	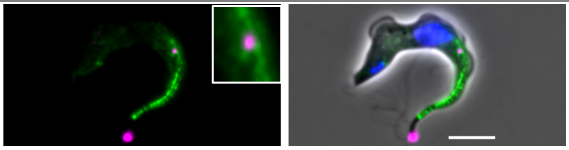	Distal FAZ, anterior cell tip, cytoplasm
Tb427.01.2260 – SMP1-2	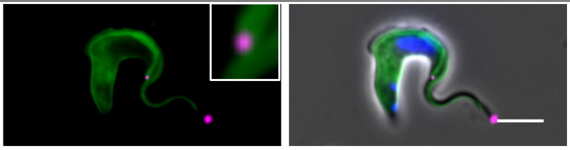	Flagellar membrane, pellicular membrane
Tb427.04.5340 – FAZ11	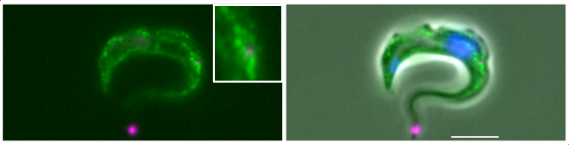	Anterior cell tip, FAZ
Tb427.08.6830	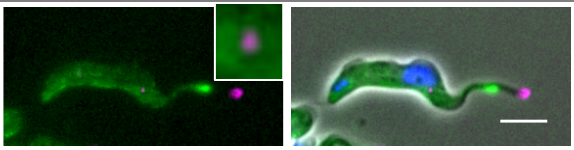	Anterior cell tip, cytoplasm

Tb427.09.8180	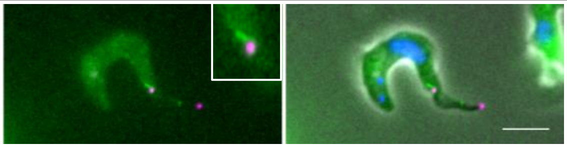	Anterior cell tip, FAZ, cytoplasm
Tb427.10.2160 – DUF1935	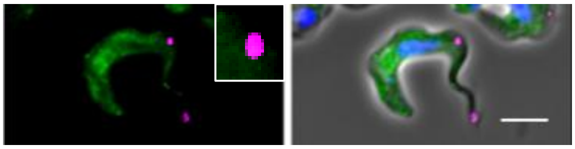	Anterior cell tip, cytoplasm
Tb427.10.15390 – FAZ7	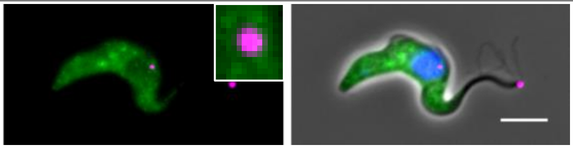	Cytoplasm
Tb427tmp.01.0200 - Katanin	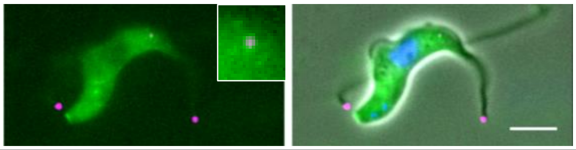	Cytoplasm
Tb427tmp.01.1050 – FAZ20	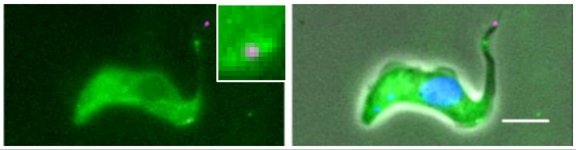	Anterior cell tip, cytoplasm
Tb427.03.4960 – FCP2	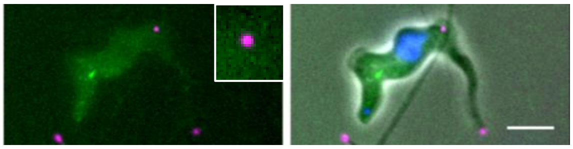	Cytoplasm
Tb427.08.960 – FCP1	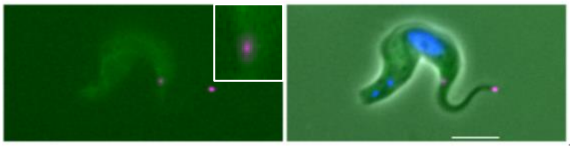	Cytoplasm, ER
Tb427.08.7540 – FCP3	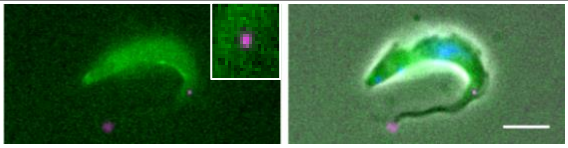	Cytoplasm
Tb427.10.9380	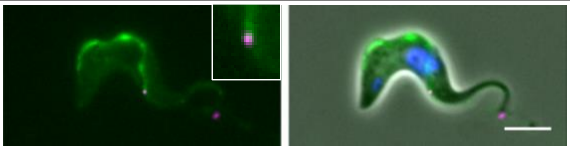	Cytoplasm, FAZ
Tb427.10.12360	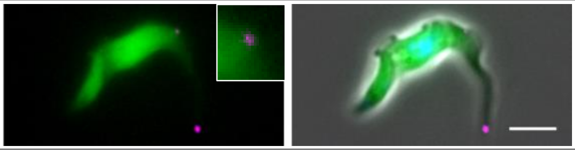	Cytoplasm
Tb427tmp.02.1000	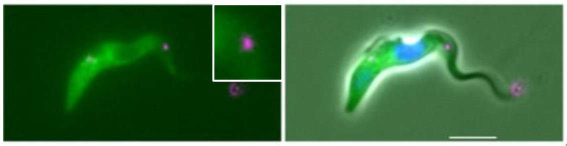	Cytoplasm
Tb427tmp.01.1680	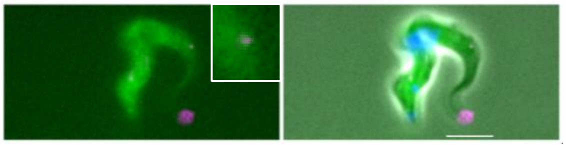	Cytoplasm

Tb427tmp.50.0007	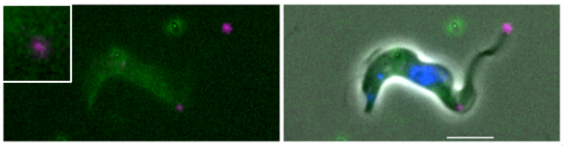	**Cytoplasm**
Tb427tmp.47.0015 – FC1	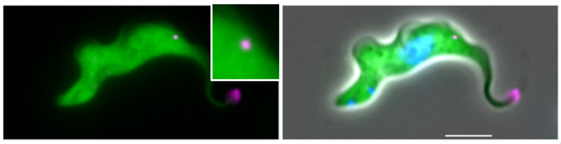	Cytoplasm
Tb427tmp.02.2050 – FCP6	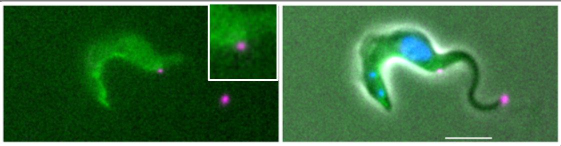	Cytoplasm
Tb427.03.4710 – FAZ23	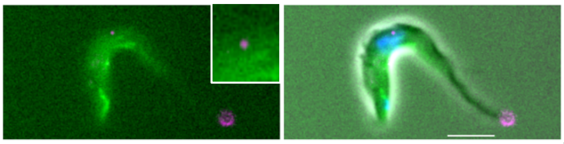	Cytoplasm
Tb427.04.2060 – FAZ8	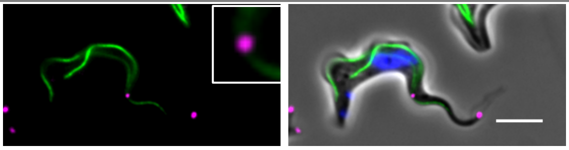	FAZ
Tb427.08.5350	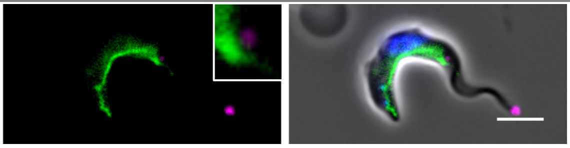	Cytoplasm
Tb427.10.7210 – FAZ17	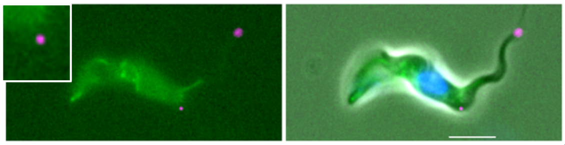	Cytoplasm
Tb427.10.12470	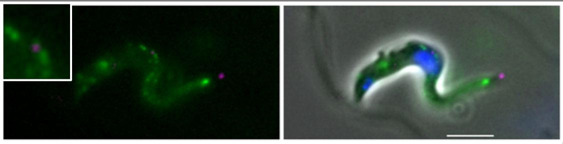	Cytoplasm, anterior cell tip
Tb427tmp.02.0890	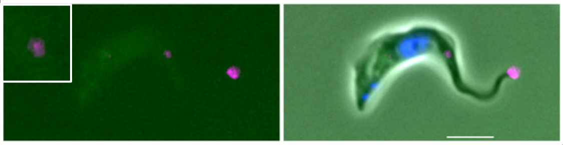	Cytoplasm
Tb427.10.5870 – FAZ25	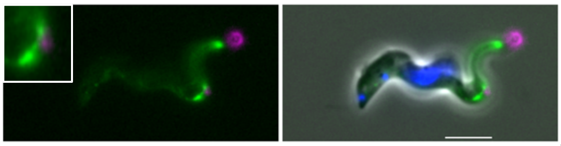	Cytoplasm, anterior cell tip

Supplementary figure 3: 32 proteins did not localise to the flagellar groove. Representative 2K1N cells of the 32 cell lines generated as part of our screen, with these proteins not localised to the flagellar groove. Scale = 5 µm.

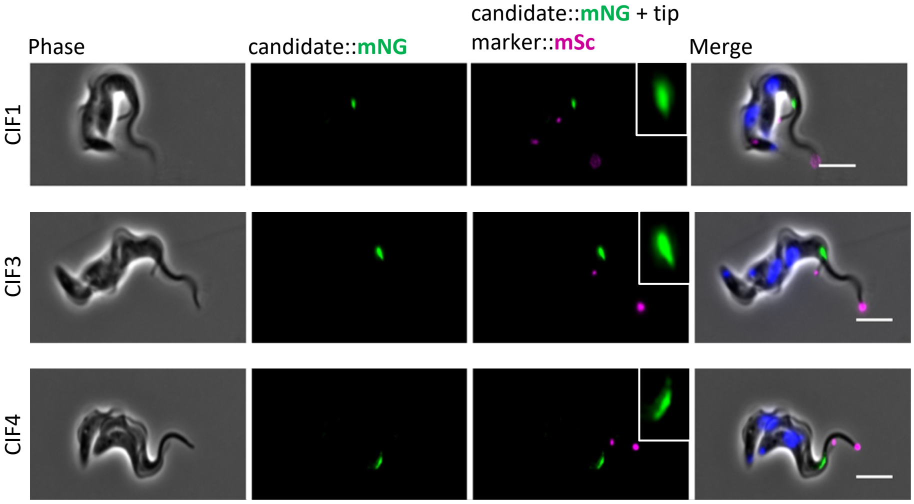

Supplementary figure 4: CIF1, CIF3 and CIF4 localised to the cleavage furrow during cytokinesis. Endogenous tagging of CIF1, CIF3 and CIF4 with mNeonGreen revealed a green foci at the site of cleavage furrow ingression (inset). Scale = 5 µm.

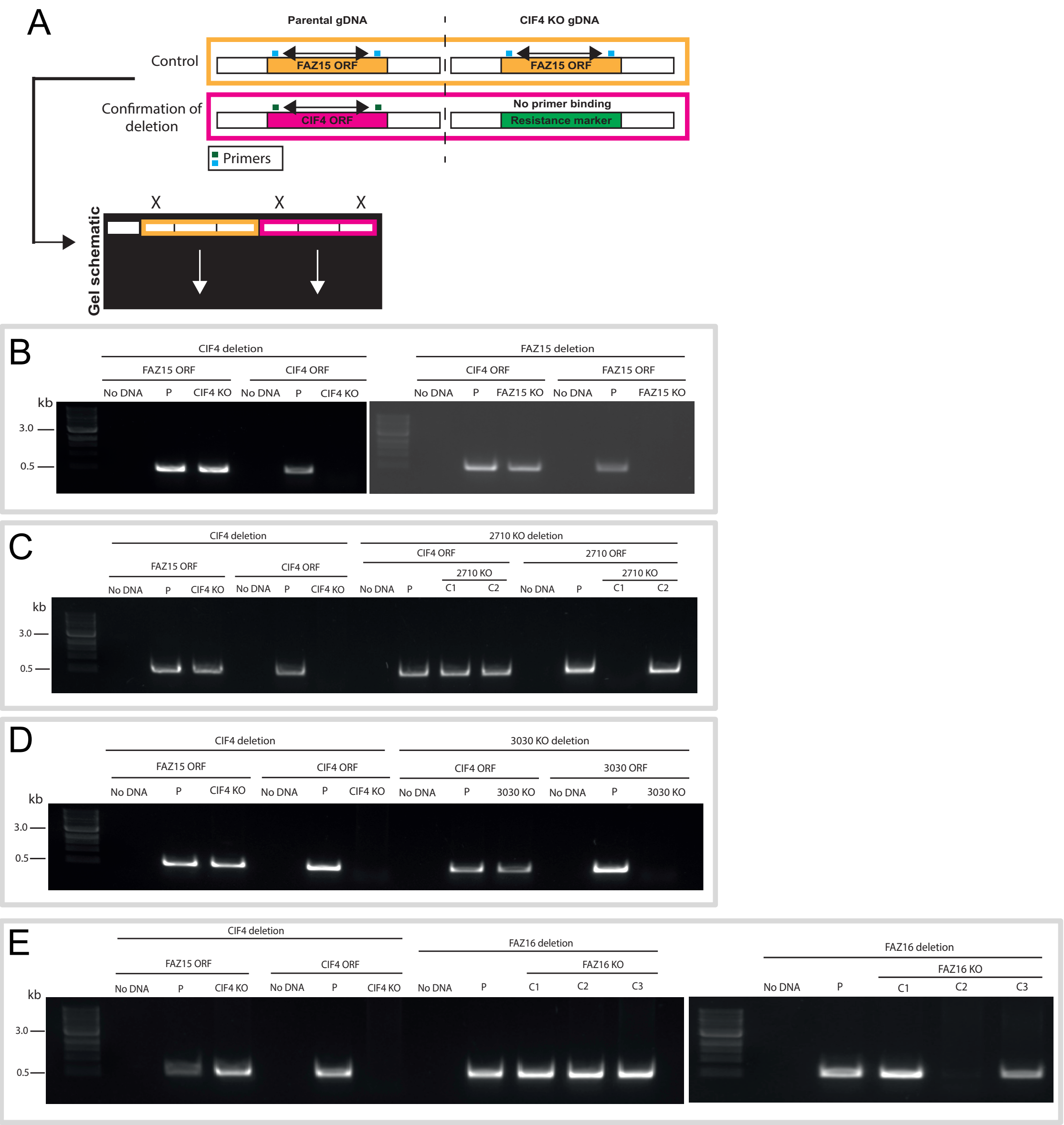

<u>Supplementary figure 5:</u> CIF4, FAZ15, FCP7 (2710), FAZ45 (3030), and FAZ16 were successfully deleted. A) Example schematic for confirming the deletion of CIF4. X denotes lanes where the reaction resulted in no DNA amplification. B) PCR confirmation of the deletion of both CIF4 (left) and FAZ15 (right). Controls for each reaction include ddH_2O (no DNA) and parental genomic DNA (P), as well as constructs that recognise another gene as a positive control. C-E) PCR confirmation of the deletion of Tb427.01.2710 clone 1 (C1), Tb427tmp.01.3030 and FAZ16. Controls include ddH_2O and parental genomic DNA (P). We also tested for the deletion of CIF4 as a control for each reaction (left of gels).

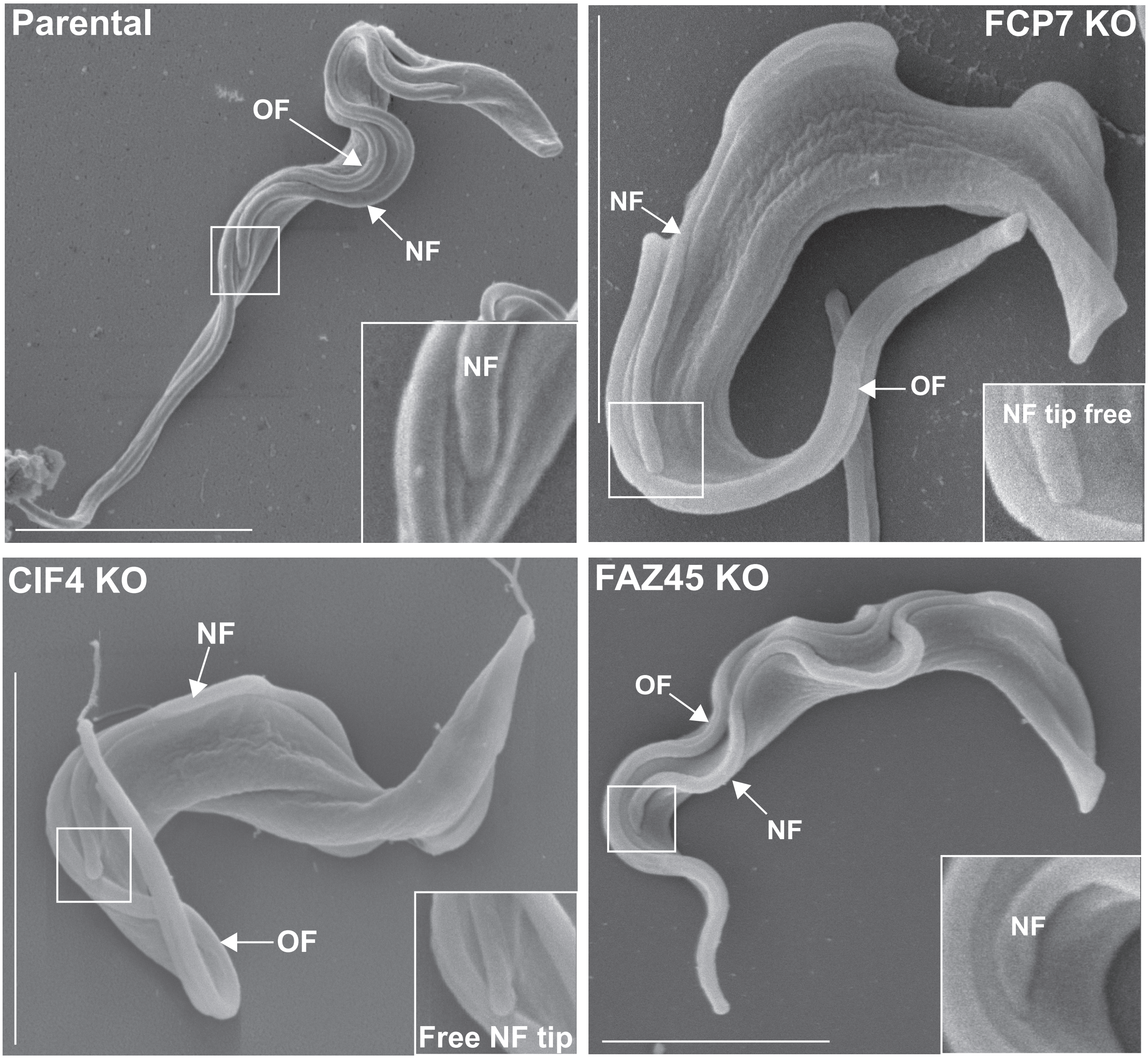

<u>Supplementary figure 6:</u> Deletion of FCP7 and CIF4 caused premature new flagellum tip detachment. In parental cells, the distal tip of the new flagellum is embedded in the cell body in a groove, however, in the CIF4 and FCP7 deletion cell lines, the new flagellum tip is prematurely released from the groove. Key: NF = new flagellum; OF = old flagellum. Scale = 5 µm.

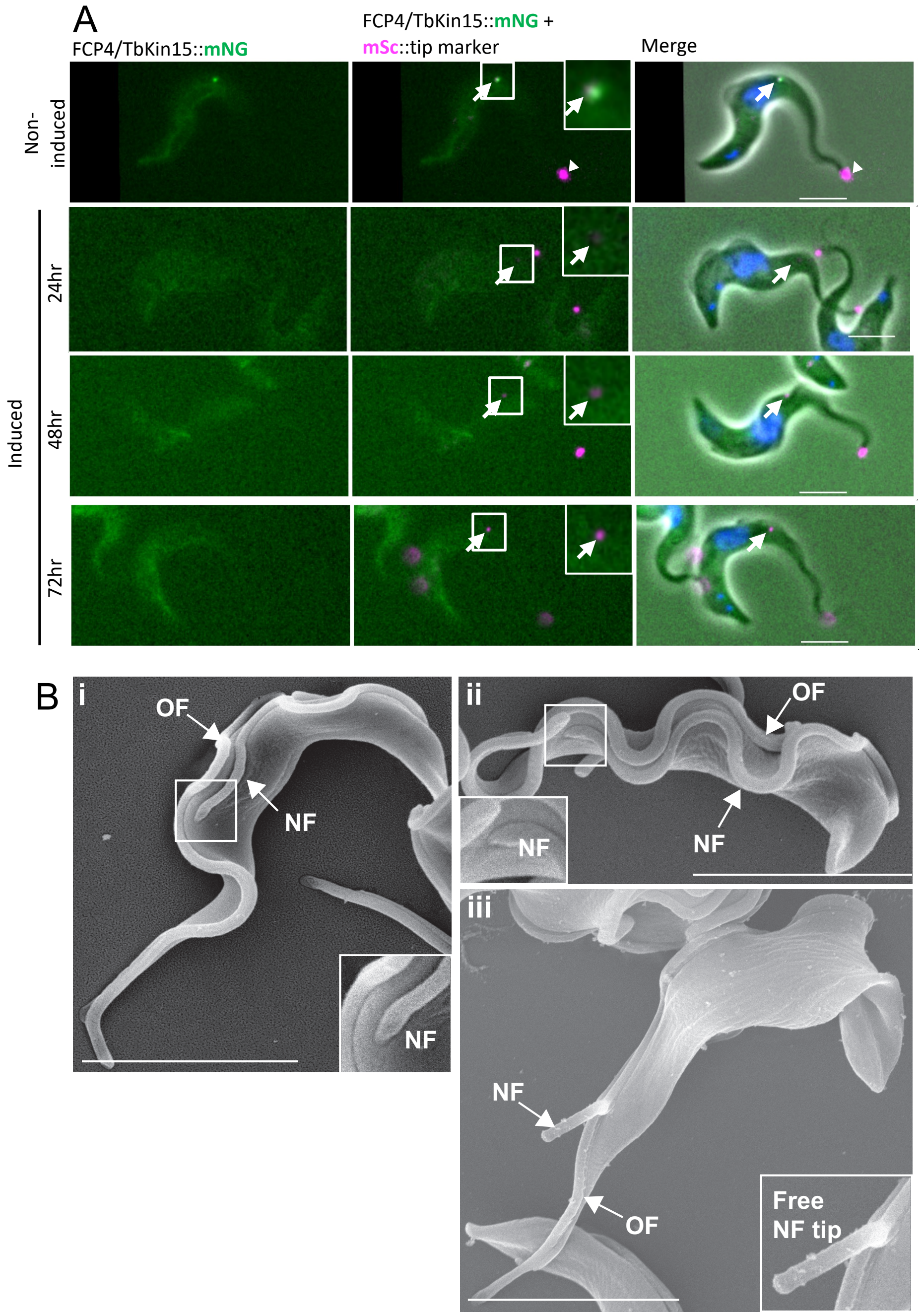

Supplementary figure 7: A) Depletion of a FCP4/TbKin15 caused a reduction in green signal after induction, showing that the RNAi was successful and was maintained throughout 72 hours. Non-induced population shows FCP4/TbKin15::mNG localisation in the groove (inset box, arrow). Induced populations show that the FCP4/TbKin15::mNG green foci has disappeared as a result of induction with doxycycline. DNA stained with Hoechst (blue). Scale = 5 µm. B) Depletion of FCP4/TbKin15 caused premature new flagellum tip detachment. i-ii) Representative cells from a non-induced population showing the distal tip of new flagellum embedded in the groove. iii) Representative cell showing new flagellum tip released from the groove 72 hours post-induction with doxycycline. Key: NF = new flagellum; OF = old flagellum. Scale = 5 µm.

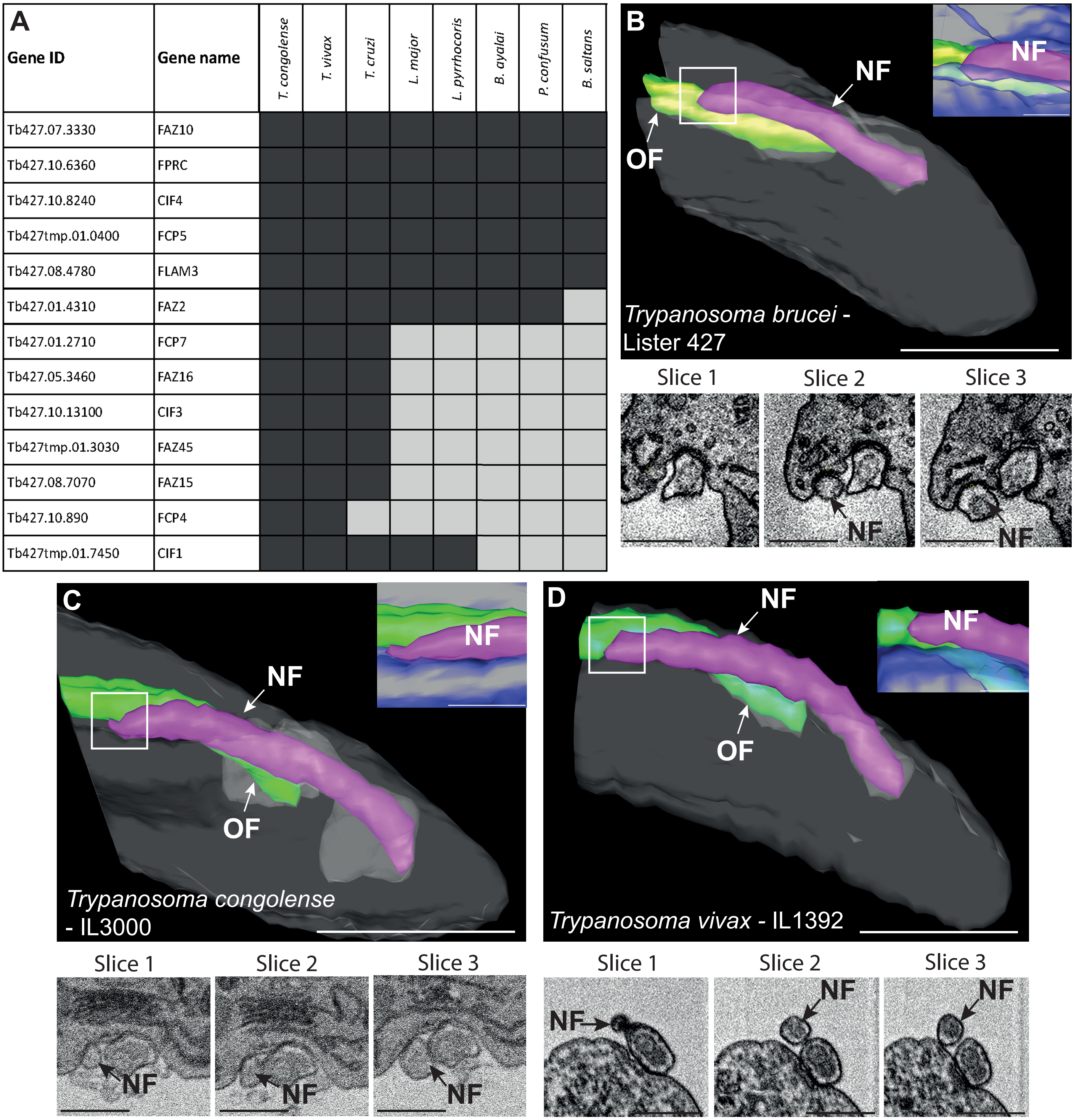

<u>Supplementary figure 8:</u> FCP7, FAZ16, CIF3, FAZ45 and FAZ15 are trypanosomatid-specific proteins, whereas FCP4/TbKin15 is conserved only in the African trypanosomatids. A) Conservation of the 13 groove proteins among related kinetoplastid organisms. Presence of orthologue indicated by dark grey shading; light grey shading indicates no orthologue identified. Expected-value cut-off = e^{-10}. Species (left to right): *Trypanosoma vivax; Trypanosoma congolense; Trypanosoma cruzi; Leishmania major; Leptomonas pyrrhocori; Blechomonas ayalai; Paratrypanosoma confusum; Bodo saltans.* B-D) Surface rendering of representative early-stage cells with a short new flagellum from *T. brucei* (Lister 427 culture strain), *T. congolense* (IL3000) and *T. vivax* (IL1392). For each strain, the new flagellum distal tip is not fully embedded in a groove when the new flagellum is short. N ≥ 3 models per species. Key: NF = new flagellum; OF = old flagellum. Scale of cell models = 2 µm; Scale of enlarged model insets and SBFSEM raw data slices = 500 nm.
